# Proteoglycan 4 (PRG4) treatment enhances wound closure and tissue regeneration

**DOI:** 10.1038/s41536-022-00228-5

**Published:** 2022-06-24

**Authors:** Roman J. Krawetz, Saleem Abubacker, Catherine Leonard, Anand O. Masson, Sophia Shah, Nadia Narendran, Pankaj Tailor, Suresh C. Regmi, Elodie Labit, Nicoletta Ninkovic, Jessica May Corpuz, Kenichi Ito, T. Michael Underhill, Paul T. Salo, Tannin A. Schmidt, Jeff A. Biernaskie

**Affiliations:** 1 McCaig Institute for Bone & Joint Health, University of Calgary, Calgary, AB Canada; 2 Biomedical Engineering Graduate Program, University of Calgary, Calgary, AB Canada; 3 Department of Surgery, University of Calgary, Calgary, AB Canada; 4 Department Cell Biology and Anatomy, University of Calgary, Calgary, AB Canada; 5 Faculty of Kinesiology, University of Calgary, Calgary, AB Canada; 6 Department of Comparative Biology and Experimental Medicine, University of Calgary, Calgary, AB Canada; 7 Department of Biochemistry and Molecular Biology, University of Calgary, Calgary, AB Canada; 8 Department of Cellular and Physiological Sciences and Biomedical Research Centre, University of British Columbia, Vancouver, BC Canada; 9 Biomedical Engineering Department, University of Connecticut Health Center, Farmington, CT USA; 10 Alberta Children’s Hospital Research Institute, University of Calgary, Calgary, AB Canada

**Keywords:** Regeneration, Mesenchymal stem cells, Cartilage

## Abstract

The wound healing response is one of most primitive and conserved physiological responses in the animal kingdom, as restoring tissue integrity/homeostasis can be the difference between life and death. Wound healing in mammals is mediated by immune cells and inflammatory signaling molecules that regulate tissue resident cells, including local progenitor cells, to mediate closure of the wound through formation of a scar. Proteoglycan 4 (PRG4), a protein found throughout the animal kingdom from fish to elephants, is best known as a glycoprotein that reduces friction between articulating surfaces (e.g. cartilage). Previously, PRG4 was also shown to regulate the inflammatory and fibrotic response. Based on this, we asked whether PRG4 plays a role in the wound healing response. Using an ear wound model, topical application of exogenous recombinant human (rh)PRG4 hastened wound closure and enhanced tissue regeneration. Our results also suggest that rhPRG4 may impact the fibrotic response, angiogenesis/blood flow to the injury site, macrophage inflammatory dynamics, recruitment of immune and increased proliferation of adult mesenchymal progenitor cells (MPCs) and promoting chondrogenic differentiation of MPCs to form the auricular cartilage scaffold of the injured ear. These results suggest that PRG4 has the potential to suppress scar formation while enhancing connective tissue regeneration post-injury by modulating aspects of each wound healing stage (blood clotting, inflammation, tissue generation and tissue remodeling). Therefore, we propose that rhPRG4 may represent a potential therapy to mitigate scar and improve wound healing.

## Introduction

While many tissues demonstrate adequate wound closure under normal circumstances, in cases where the primary insult (injury/disease) persists, this can result in chronic inflammation and inappropriate fibrotic repair. Chronic inflammation prevents closure, as evident in ulcerative lesions. While continued fibrosis in the skin leads to scarring and disfigurement, progressive deposition of extracellular matrix in organs compromises their structure and function, causing disease and even death^1^.

Full-thickness skin wounds (both acute and chronic) have a tremendous impact on quality of life, affecting 6.5 million patients in North America, and costing an excess of $25 billion annually to treat^2^. While numerous therapeutic approaches have been developed in an attempt to treat full-thickness wounds, such as surgical intervention (grafts), pharmacological therapies and rehabilitation, outcomes remain poor and often with life-long disbality^3^. It is widely agreed that inducing tissue regeneration and/or enhancing endogenous repair would be an ideal therapeutic approach for chronic wounds^4–6^. While previous studies have demonstrated that application of small molecules can enhance tissue regeneration^7^, in many cases, the molecular pathways that these drugs regulate are not fully understood and many clinical trials are unable to demonstrate treatment efficacy with potential hurdles including patient specific factors such as epigenetic status and/or medical co-morbidities. In additional to chronic wounds, mammals typically do not demonstrate cartilage repair/regeneration post-injury. There are a few notable exceptions such as the African Spiny mouse^8^, MRL mouse strain^9^ and p21^−/−^^10^ have the capacity to regenerate articular cartilage after a focal defect. Although mouse pinna/auricular cartilage is elastic cartilage, similar to articular cartilage, auricular cartilage does not spontaneously heal after injury^11^. While these mice and others demonstrate increased wound healing (including cartilage) after injury, these mice have a number of differences at the genetic and epigenetic levels compared to non-healing strains (such as C57BL/6 mice)^12^, which makes it difficult to determine which gene(s) are responsible for the healer phenotype.

Proteoglycan 4 (PRG4/lubricin) is found throughout the animal kingdom from fish to elephants^13^. In humans, it is found in high concentrations in the synovial fluid of joints (~400 µg/mL), and in lower concentrations (<100 µg/mL) in blood^13,14^. PRG4 reduces friction between load bearing surfaces^15,16^, and therefore, research on its potential benefits has been primarily focused on the joint; however, PRG4 is expressed in liver, heart, lung, kidney and other tissues^13^, yet its role in these tissues remains elusive. The disease Camptodactyly Arthropathy Coxa Vara Pericarditis is linked to mutations in the *Prg4* gene^17^, and these patients suffer from joint degeneration, chronic inflammation, and pericarditis^18^. This suggests that PRG4 plays key roles in tissue homeostasis and immune modulation in addition to lubrication. Recently, we demonstrated that PRG4 regulates the inflammatory response through toll-like receptors (TLRs)^19,20^, and recombinant human (rh)PRG4 treatment after injury reduces inflammation and pain in a rat model of osteoarthritis^19^. Recent studies have also expanded our knowledge of PRG4, demonstrating that PRG4 expression is required for joint formation during BMP-9-mediated digit regeneration (yet, is not required for joint formation during normal development)^21^ and that it can also regulate the expression of genes associated with the fibrotic response^22^. These results and others are widening our view on PRG4 signalling and suggest that this protein may be a potent regulator of tissue repair and/or regeneration. Therefore, in the current study we aimed to determine if exogenously delivered PRG4 can enhance connective tissue regeneration after injury using a mouse ear wound assay.

## Results

### rhPRG4 treatment accelerates ear wound closure

**Fig. 1:**
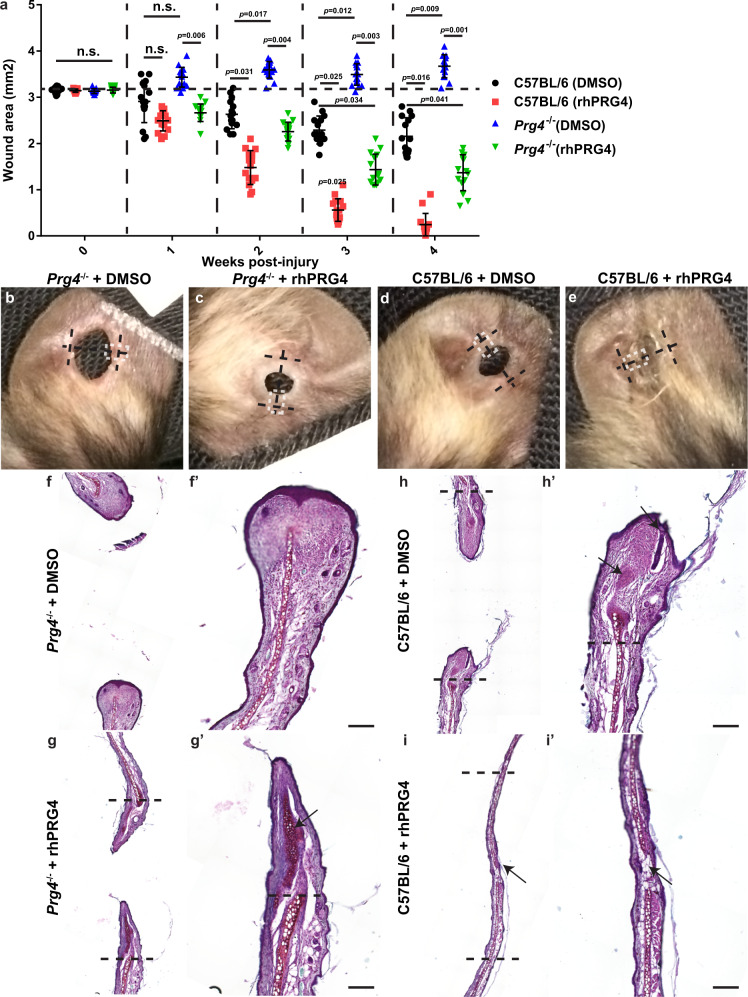
rhPRG4 simulates regeneration of ear wound injury. rhPRG4 treatment results in near total closure of the ear wound 4 weeks-post injury, while *Prg4*^−/−^ demonstrate no wound closure. This is rescued by the addition of rhPRG4 **a**. Photographs of the representative ears 4 weeks post-injury **b**–**e**. The black dashed line represents the plane of histological sectioning, the parallel black bars represent the original injury diameter **b**–**e**, while the white dashed box indicates the location of the histological image presented **b**–**e**. Representative histological images stained with Safranin O, 4 weeks post-injury **f**–**i**. Mainly fibrotic-like tissue was observed in the injury site of C57BL/6 and *Prg4*^−/−^ mice treated with DMSO **f**, **h**. In *Prg4*^−/−^ mice treated with rhPRG4, new cartilage like-tissue can be observed in the injury site (arrow g). New cartilage bridging the injury site can be observed in C57BL/6 mice treated with rhPRG4 and only small areas are present that still lack cartilage (arrows i). The black dashed line represents site of original injury Scale bar equals 50 µm. n.s. = not significant. Sample sizes: a-i, *n* = 15 per group. Error bars equal mean ± SD (a - 2 way ANNOVA).

While auricular cartilage injuries do not completely close in mice, there are healer strains such as the MRL and *p21*^−/−^ which can regenerate post-injury^23,24^. C57BL/6 mice are a non-healer strain, in which auricular injuries close through fibrotic-like repair^12^. Therefore, we examined the healing response in *Prg4*^−/−^ mice (Fig. 1a, b, f). The normal wound closure response was abrogated, with the wound area actually appearing to increase over time (Fig. 1a, b, Supplementary Fig. 2). Furthermore, the receding tissue was found to be mainly comprised of a fibrous-like tissue (Fig. 1f). To determine if exogenous delivery of PRG4 could rescue this phenotype, the injured *Prg4*^−/−^ mice were treated with rhPRG4 (Fig. 1a, c, g) and increased wound closure was observed in comparison to *Prg4*^−/−^ mice. Furthermore, in *Prg4*^−/−^ mice treated with rhPRG4, formation of both fibrous-like tissue and auricular-like cartilage (Fig. 1g, arrow) was observed within the injury site. Since it appeared that *Prg4* was required for the normal healing response, it was next examined if exogenous application of rhPRG4 could promote enhanced healing. As expected, 2 mm punch biopsy injuries through the auricular cartilage in C57BL/6 mice treated with DMSO alone, demonstrated minimal wound closure by 4-weeks post-injury (Fig. 1a, d, h, Supplementary Fig. 2), with fibrous-like tissue observed in the injury site (Fig. 1h arrows). In contrast, C57BL/6 mice treated with 100 µg/mL rhPRG4 (in 10 µl DMSO) showed almost complete wound closure and presented with a near completely regenerated auricular-like cartilage pinnae within the injury site (Fig. 1a, e, i arrow). Haematoxylin and eosin staining of all groups are also presented in Supplementary Fig. 1. Since the rate of ear wound closure was slightly decreased in C57BL/6 mice treated with DMSO compared to the published literature, we also examined untreated (no DMSO nor rhPRG4) injuries in C57BL/6 and *Prg4*^−/−^ mice (Supplementary Fig. 3). C57BL/6 and *Prg4*^−/−^ mice demonstrated improved wound closure in the absence of DMSO treatment, yet *Prg4*^−/−^ mice still displayed a significant healing impairment compared to C57BL/6 mice (Supplementary Fig. 3). This observation is consistent with the published literature since DMSO can reduce scaring post-injury^25^ and these auricular injuries normally close through fibrotic-like repair.

Since PRG4 is historically known as a cartilage lubrication molecule and the effect of DMSO on PRG4 lubricating ability is unknown, it was decided to test if DMSO impacted PRG4 lubrication and by extension determine if tissue/auricular cartilage lubrication could be playing a role in the enhanced wound healing observed with application of rhPRG4 (Supplementary Fig. 3). It was observed that rhPRG4 displayed increased lubricating ability compared to PBS (negative control) and DMSO (Supplementary Fig. 3). Yet, when rhPRG4 was diluted in DMSO (vs. the same concentration in ‘PBS – rhPRG4’ lane), its lubricating ability was lost suggesting the effects observed in tissue repair were not due to lubrication.

### rhPRG4 treatment increases blood flow at the injury site

**Fig. 2:**
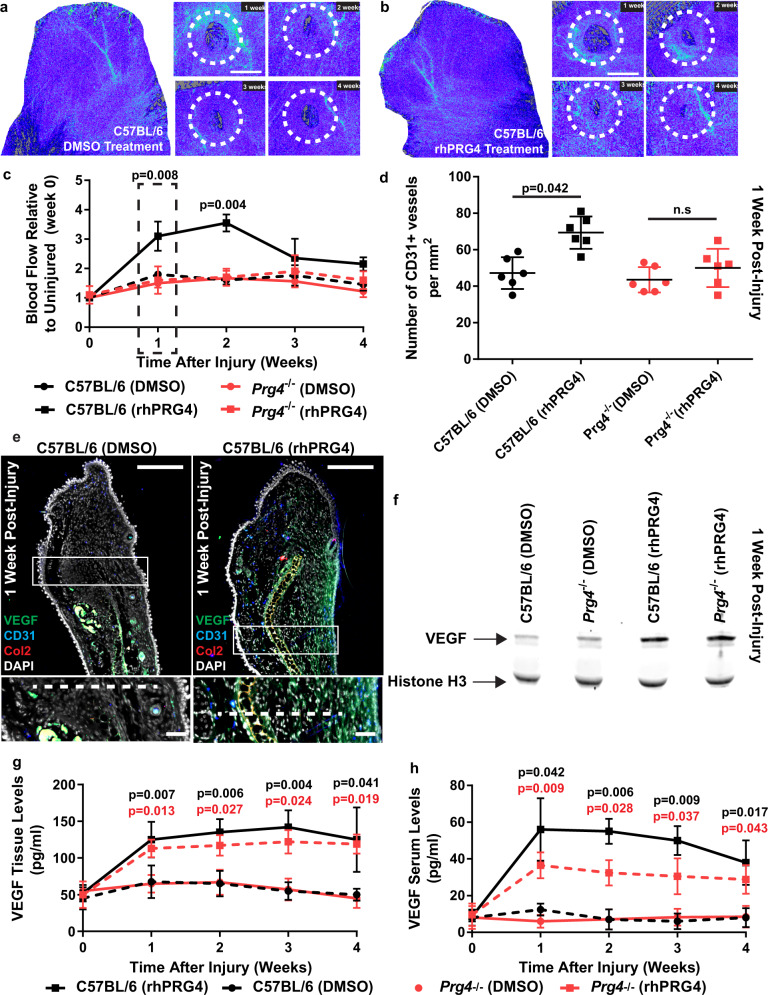
rhPRG4 treatment post-injury increases blood flow and VEGF expression. Representative images of blood flow captured through laser refraction speckle imaging, with lighter shades of blue equaling higher flow rate **a**, **b**. Quantitative analysis of blood flow data demonstrates that C57BL/6 mice treated with rhPRG4 show increased blood flow at the wound area post-injury **c**. The number of CD31 + blood vessels also increased in the wound area site with rhPRG4 treatment **d**. Immunostaining of the injury site for VEGF, CD31 and Collagen 2 demonstrated increased expression of all three proteins with rhPRG4 treatment **e**. VEGF levels were confirmed by western blot analysis using a tissue homogenate from the injury site, demonstrating increased VEGF expression (~39 kDa) with Histone H3 as a loading control (~18 kDa) **f**. Local (injury site) and systemic (serum) levels of VEGF were quantified over time post-injury by Luminex and both were found to increase with local rhPRG4 treatment **g**, **h**. Scale bar equals 2000 µm **a**, **b**; 50 µm (top panels), 10 µm (bottom panels) **e**. n.s. = not significant. Error bars equal mean ± SD (**c**, **g**, **h** 1 way ANNOVA, **d** – t-test). Sample sizes: **a**, **b**, **c**, **h**, *n* = 10 per group; **d**, **e**, **f**, **g**, *n* = 6 per group.

To investigate the mechanism by which PRG4 accelerates wound closure and promotes cartilage regeneration, we first examined blood flow to the injury site for a number of reasons. We have recently shown that rhPRG4 is able to induce the expression of VEGF in human synovial fibroblasts^26^; we also observed that in mice treated with rhPRG4 the wound area appeared to bleed more than DMSO controls at earlier timepoints (within the first week post-injury); and rhPRG4 treated ears were significantly warmer (*p* = 0.042)(39.5 ± 0.6 °C SD, *n* = 10) than DMSO treated injuries (38.2 ± 0.6 °C SD, *n* = 10) and non-injured ears (37.7 ± 0.3 °C SD, *n* = 10). Therefore, blood flow in and around the injury site was visualized and quantified using laser speckle contrast imaging, with mice being randomized between DMSO and rhPRG4 treatment groups. Mice in the rhPRG4 treatment group appeared to have increased and sustained blood flow within and adjacent to the injury site (Fig. 2a, b). The blood flow data was quantified, and it was found that rhPRG4 treated C57BL6 mice demonstrated increased blood flow at the injury site at 1- and 2-week post-injury with no differences from DMSO treated mice by 3- and 4-weeks post-injury (Fig. 2c). *Prg4*^−/−^ mice treated with DMSO demonstrated similar levels to C57BL/6 mice treated with DMSO. Interestingly, *Prg4*^−/−^ mice treated with rhPRG4 did not demonstrate any increase in blood flow compared to *Prg4*^−/−^ or C57BL/6 mice treated with DMSO (Fig. 2c). To further explore this result, the number of CD31^+^ blood vessels were quantified in the injury site at 1-week post-injury and the same pattern as the blood flow data was observed. rhPRG4 treated C57BL/6 injuries demonstrated increased CD31^+^ blood vessels, while there were no differences between C57BL/6 mice treated with DMSO and *Prg4*^−/−^ mice treated with DMSO or rhPRG4 (Fig. 2d). Ear injuries were also examined histologically and for expression of CD31, VEGF and Col2 at 1-week post-injury (Fig. 2e, Supplementary Fig. 4). In addition to an increase in CD31 staining, an increase in VEGF staining was observed. To determine if VEGF protein levels were increased in the injury site with rhPRG4 treatment, the ear injury site was biopsied and probed for VEGF expression using western blot and Luminex analysis. While VEGF was detectable in all samples, it was increased in mice treated with rhPRG4 (Fig. 2f, g Supplementary Fig. 5). Interestingly, VEGF was also found to be increased in *Prg4*^−/−^ mice treated with rhPRG4 even though these mice did not demonstrate an increase in blood flow nor CD31^+^ staining. When the VEGF protein levels were quantified by Luminex, it was found that rhPRG4 treatment significantly increased VEGF levels at the injury site in both C57BL/6 and *Prg4*^−/−^ mice (Fig. 2g). Furthermore, when serum levels of VEGF were quantified by Luminex, the same trend was observed demonstrating that local rhPRG4 treatment induced a systemic response, however, the VEGF response was muted in *Prg4*^−/−^ mice (Fig. 2h).

### rhPRG4 induces VEGF expression through a TLR-dependent mechanism

**Fig. 3:**
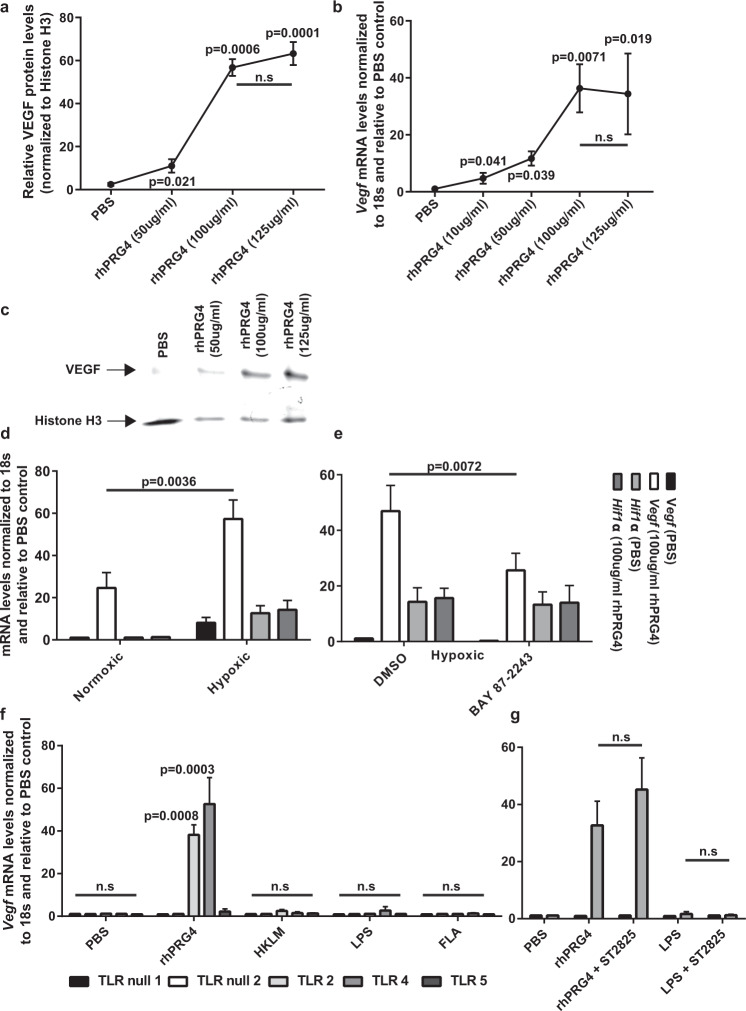
rhPRG4 induces VEGF expression in vitro. In primary MEFs, rhPRG4 induced VEGF protein expression in a dose dependent manner with a plateau at 100 µg/ml **a**, **b**. This effect was confirmed at the mRNA level **c**. *Vegf* and *Hif1α* mRNA expression was quantified under normoxic and hypoxic conditions in the presence or absence of rhPGR4 and/or Hif1α inhibition (BAY 87-2243) to demonstrate that rhPRG4 induction of *Vegf* was independent of Hif1α **d**, **e**. To determine if *Vegf* activation was downstream of Toll-like receptors (TLRs), HEK cells lacking all TLRs or expressing a single TLR (2, 4 or 5) were treated with rhPRG4 or their specific agonist **f**. TLR4 expressing cells demonstrated the maximal *Vegf* activation (f) and this activation was independent of MYD88 activity since the MYD88 inhibitor (ST2825) had no effect on rhPRG4 *Vegf* activation **g**. All experiments were undertaken on at least 3 biological and 3 technical replicates unless otherwise stated. n.s. = not significant. Error bars equal mean ± SD (**a**, **c**, **d** – 1 way ANNOVA, **f**, **g** – t-test).

Primary mouse embryonic fibroblasts (MEFs) derived from C57BL/6 mice were exposed to increasing levels of rhPRG4 in vitro and VEGF was assayed at the protein (Fig. 3a, b) and transcript (Fig. 3c) levels. VEGF was expressed at baseline (protein and mRNA) levels in the absence of rhPRG4 and increased in a dose-response fashion within increasing rhPRG4 concentrations. However, there was a plateau of VEGF expression post-rhPRG4 treatment, with no increase in VEGF protein or *Vegf* transcript observed with over 100 µg/mL rhPRG4 (Fig. 3a, b, Supplementary Fig. 5). Since VEGF is known to be downstream of HIF1α and is upregulated under hypoxic conditions, we investigated if rhPRG4 induced *Vegf* through *Hif1α* and if there was any synergy if the cells were cultured under hypoxic (vs. normoxic) conditions (Fig. 3d). In MEFs grown in normoxic conditions, rhPRG4 induced *Vegf* expression, but not *Hif1α* expression (Fig. 3d), while under hypoxic conditions, both *Vegf* and *Hif1α* were upregulated (Fig. 3d). Furthermore, there was a synergistic effect of rhPRG4 and hypoxia on *Vegf* expression, yet no effect was observed on *Hif1α* expression (Fig. 3d). Since HIF1α can induce *Vegf* expression without the requirement for an increase in *Hif1α* expression, we sought to determine if HIF1α was required for rhPRG4 mediated *Vegf* upregulation by treating MEFs under hypoxic conditions with a HIF1α inhibitor (BAY 87-2243). While BAY 87-2243 treatment had no effect on *Hif1α* expression, it significantly inhibited rhPRG4 induced *Vegf* levels (Fig. 3e), comparable to rhPRG4 induced *Vegf* levels under normoxic conditions (Fig. 3d). While these results suggest that rhPRG4 induction of *Vegf* can be enhanced by hypoxia, this mechanism is not fully dependent on hypoxia and/or hypoxia induced *Hif1α*. There are many other signalling mechanisms other than hypoxia that can induce *Vegf*, one of these is Toll-like receptor (TLR) signalling^27^. We have previously reported that rhPRG4 can bind to and activate pathways downstream of TLR2, 4 and 5^19^, and therefore examined if rhPRG4 could induce *Vegf* through TLR signalling (Fig. 3f). Neither, PBS, HKLM, LPS nor FLA was able to induce *Vegf* mRNA, yet, rhPRG4 was able to induce *Vegf* mRNA in TLR2 and 4 expressing cells, but not in TLRnull (1/2) or TLR5 expressing cells (Fig. 3f). To determine if rhPRG4 induction of *Vegf* mRNA was Myd88 dependent, TLRnull (1) and TLR4 reporter lines were treated with the Myd88 inhibitor ST2825^28^ and exposed to rhPRG4 or LPS (Fig. 3g). No induction of *Vegf* mRNA was observed in TLRnull cells treated with rhPRG4 (w/o ST2825), or in TLRnull or TLR4 cells treated with LPS (with/without ST2825). Confirming previous results, rhPRG4 induced *Vegf* mRNA in TLR4 cells and this was not inhibited by ST2825, suggesting this pathway is Myd88 independent (Fig. 3g).

### *Tlr4*^*−/−*^ mice demonstrate increased wound closure that can be enhanced with rhPRG4

**Fig. 4:**
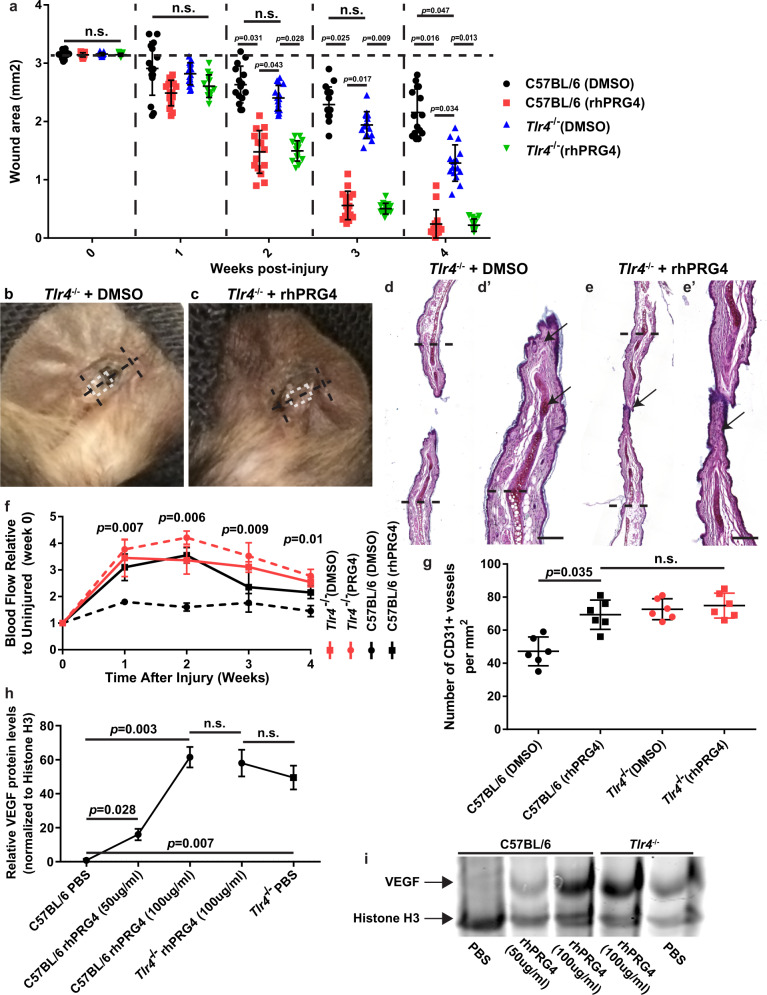
*Tlr4*^*−/−*^ mice demonstrate increased wound healing and blood flow post-injury. *Tlr4*^−/−^ demonstrate increased wound closure compared to C57BL/6 mice. This is enhanced in the presence of rhPRG4 **a**. Representative images of ears 4 weeks post-injury **b**, **c**. The black dashed line represents the plane of histological sectioning which the original injury diameter, while the white dashed box indicates the location of the histological image presented **b**, **c**. Histological images stained with Safranin O, 4 weeks post-injury **d**, **e**, with the black dashed line representing the original injury site. New immature cartilage can be observed in *Tlr4*^−/−^ mice treated with DMSO (arrows, **d**). Although the wound is nearly closed, minimal cartilage islands are observed in *Tlr4*^−/−^ mice treated with rhPRG4 (arrow **e**). Quantitative analysis of blood flow data demonstrates that *Tlr4*^−/−^ mice treated with DMSO or rhPRG4 show increased blood flow at the wound area post-injury **f**. The number of CD31 + blood vessels are also increased in the wound area site of *Tlr4*^−/−^ mice (g). MEFs from *Tlr4*^−/−^ mice demonstrate higher baseline levels of VEGF and do not increase the in presence of rhPRG4 **h**, **i**. Scale bar equals 500 µm. n.s. = not significant. Error bars equal mean ± SD (**f**–**h** – 1 way ANNOVA, a – 2 way ANNOVA). Sample sizes: **a**–**f**, *n* = 15 per group; **g**–**i**, *n* = 6 per group.

To determine if TLR4 is involved with rhPRG4 induced tissue regeneration, *Tlr4*^−*/*−^ mice were subjected to 2 mm biopsy injuries through the auricular cartilage and treated with rhPRG4 or DMSO vehicle alone (Fig. 4a–c). *Tlr4*^−*/*−^ mice treated with DMSO demonstrated increased wound closure compared to C57BL/6 mice (Fig. 4a, b, Supplementary Fig. 6) whereas *Tlr4*^−*/*−^ mice treated with rhPRG4 demonstrated near identical wound closure to C57BL/6 mice treated with rhPRG4 (Fig. 4a, c, Supplementary Fig. 6). Histologically, in *Tlr4*^−*/*−^ mice treated with DMSO, both fibrous-like tissue and auricular-like cartilage were observed within the injury site (Fig. 4d), yet the cartilage like-tissue observed within the injury had not fully matured (Fig. 4d, arrow). This contrasts with C57BL/6 mice treated with DMSO that presented with primarily fibrous-like tissue observed in the injury site (Fig. 1f). *Tlr4*^−*/*−^ mice treated with rhPRG4 showed almost complete wound closure yet presented with minimal auricular-like cartilage within the distal margins of the injury site (Fig. 4e, arrow) and the cartilage-like tissue within the injury site appeared immature. *Tlr4*^−*/*−^ mice demonstrated increased blood flow (Fig. 4f) and increased number of CD31^+^ blood vessels (Fig. 4g) within the wound site regardless of rhPRG4 treatment, suggesting that a pathway downstream of TLR4 activation normally inhibits this mechanism and rhPRG4 binding to TLR4, or the absence of TLR4, is sufficient to increase blood flow/angiogenesis. To confirm this, primary mouse embryonic fibroblasts (MEFs) were derived from C57BL/6 and *Tlr4*^−*/*−^ mice and treated with rhPRG4 (100 µg/ml) or PBS and VEGF levels were assayed by western blot and quantified (Fig. 4h, i). While C57BL/6 MEFs demonstrated a positive dose response in VEGF expression due to rhPRG4 treatment, *Tlr4*^−*/*−^ MEFs expressed higher baseline levels of VEGF but were unchanged in response to rhPRG4 treatment (Fig. 4h, i, Supplementary Fig. 5).

### *Pai1*^*−/−*^ mice demonstrate increased wound closure that is enhanced by rhPRG4

**Fig. 5:**
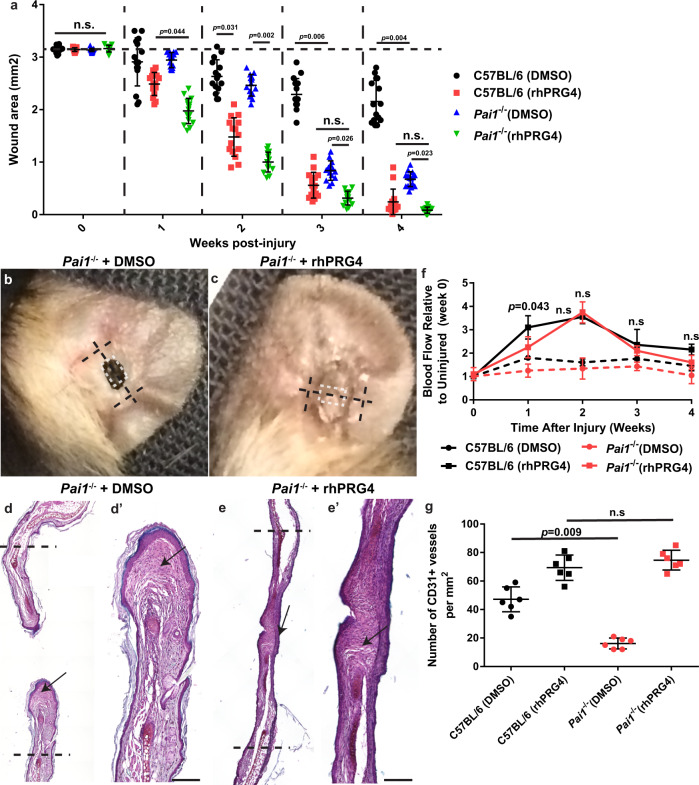
*Pai1*^*−/−*^ mice demonstrate increased wound healing post-injury. *Pai1*^−/−^ mice demonstrate increased wound closure compared to C57BL/6 mice and this is enhanced in the presence of rhPRG4 **a**. Representative images of ears 4 weeks post-injury **b**, **c**. The black dashed line represents the plane of histological sectioning which the original injury diameter, while the white dashed box indicates the location of the histological image presented **b**, **c**. Representative histological images stained with Safranin O, 4 weeks post-injury **d**, **e**. Minimal new cartilage can be observed in *Pai1*^−/−^ mice treated with DMSO (arrows, **d**). Although the wound completely closed in *Pai1*^−/−^ mice treated with rhPRG4, new cartilage is absent from the wound area (arrow **e**) yet is present at the injury boundary. Quantitative analysis of blood flow data demonstrates that *Pai1*^−/−^ mice treated with DMSO have no increase in blood flow at the wound area post-injury **f**. Furthermore, the number of CD31 + blood vessels is decreased in the wound area site of *Pai1*^−/−^ mice **g**. Scale bar equals 50 µm. n.s. = not significant. Error bars equal mean ± SD (**f**, **g** – 1 way ANNOVA, a – 2 way ANNOVA). Sample sizes: a-f, n = 15 per group; **g**–**i**, *n* = 6 per group.

Since we observed a lack of fully matured auricular cartilage within the injury site of *Tlr4*^−*/*−^ mice treated with rhPRG4, additional pathways must be regulated by rhPRG4 to obtain the regeneration observed in C57BL/6 mice treated with rhPRG4. We prioritized pathways implicated in the clotting and/or fibrotic aspects of wound healing since both mechanisms appear to be impacted by rhPRG4 treatment in wounded C57BL/6 mice. While PRG4 has no effects on the clotting of blood ex vivo^29^, PRG4 shares a number of structural similarities with Vitronectin at the N and C termini (including SMB and PEX domains)^30^. Vitronectin is known to participate in clot stabilization through binding to PAI1 and it has been previously shown that PRG4 can bind to PAI1^31^. Therefore, we asked whether a rhPRG4 - PAI1 interaction might play a role in rhPRG4 mediated tissue regeneration. *Pai1*^−*/*−^ mice received 2 mm biopsy injuries through the auricular cartilage and treated with rhPRG4 or DMSO alone (Fig. 5a–c, Supplementary Fig. 7). Similar to *Tlr4*^−*/*−^ mice, *Pai1*^−*/*−^ mice treated with DMSO showed increased wound closure compared to C57BL/6 mice (Fig. 5a, b, Supplementary Fig. 7) and *Pai1*^−*/*−^ mice treated with rhPRG4 demonstrated complete wound closure (Fig. 5a, c, Supplementary Fig. 7). However, like *Tlr4*^−*/*−^ mice, *Pai1*^−*/*−^ mice (treated with DMSO or rhPRG4) presented with minimal fully matured auricular-like cartilage within the proximal margins of the injury site (Fig. 5d, e, arrows). However, unlike *Tlr4*^−*/*−^ mice, *Pai1*^−*/*−^ mice treated with DMSO did not demonstrate any increase in blood flow (Fig. 5f) and no change in the number of wound-associated CD31^+^ blood vessels (Fig. 5g).

### PAI1 binds to rhPRG4 and may regulate the fibrotic response post-injury

Since *Pai1*^−*/*−^ mice demonstrated increased wound closure, the mechanism behind this observation was investigated. Through SPR, it was confirmed that rhPRG4 binds PAI1 (Supplementary Fig. 8a, b) with an equilibrium disassociation constant (K_D_) of 2.7^E-6^ M. To determine if rhPRG4 was regulating the fibrotic response post-injury, αSMA staining done in C57BL/6 mice treated with rhPRG4 or DMSO and compared to *Pai1*^−*/*−^ mice treated with DMSO (Supplementary Fig. 8c–n). It appeared that C57BL/6 mice treated with DMSO presented with elevated αSMA staining vs. rhPRG4 treated C57BL/6 mice and *Pai1*^−*/*−^ mice treated with DMSO (Supplementary Fig. 8c–n). The αSMA staining was quantified at 1–4 weeks post-injury and C57BL/6 mice treated with DMSO had greater αSMA staining than all other groups at all timepoints examined (Supplementary Fig. 8o). Furthermore, there was no difference in αSMA staining between C57BL/6 mice treated with rhPRG4 and *Pai1*^−*/*−^ mice treated with DMSO except at the 2 week injury timepoint with *Pai1*^−*/*−^ mice presenting with less αSMA staining (Supplementary Fig. 8o). To further explore the consequence rhPRG4-binding on fibrosis, an in vitro contraction assay was employed (Supplementary Fig. 9). C57BL/6 MEFs were loaded into collagen gels with DMSO, rhPRG4, rhPAI1 or rhPRG4 mixed with rhPAI1. DMSO had no effect on contraction, and PAI1 induced contraction as previously reported^32^. However, we were surprised to find that rhPRG4 stimulated contraction. Even more surprising, was that when rhPRG4 and PAI1 were mixed and loaded into the gel, contraction was significantly reduced compared to rhPRG4 or PAI1 alone (Supplementary Fig. 9).

### Synergism between PAI1 and TLR4 pathways in rhPRG4 mediated cartilage regeneration

Since the PAI1 and TLR4 pathways were both implicated in rhPRG4 mediated wound closure and cartilage regeneration post-injury, an examination of *Tlr4*^−*/*−^*Pai1*^−*/*−^ double knockout mice were undertaken to determine if inhibition of both pathways simultaneously could phenocopy the effects observed in C57BL/6 mice treated with rhPRG4. *Tlr4*^−*/*−^*Pai1*^−*/*−^ double knockout mice demonstrated robust wound closure ability with complete wound closure observed by 4 weeks post-injury when treated with DMSO or rhPRG4 (Supplementary Fig. 10a–c). However, *Tlr4*^−*/*−^*Pai1*^−*/*−^ mice treated with DMSO did not present with robust auricular cartilage regeneration within proximal margins of the wound (Supplementary Fig. 10d, arrows). Interestingly, and similar to what was observed with *Tlr4*^−*/*−^ and *Pai1*^−*/*−^ knockout mice, *Tlr4*^−*/*−^*Pai1*^−*/*−^ mice treated with rhPRG4 did present with auricular cartilage regeneration in the centre of the injury site, however, similar to both *Tlr4*^−*/*−^ and *Pai1*^−*/*−^ knockout mice, this cartilage was immature (Supplementary Fig. 10e, arrows). This suggests that there is interaction between the TLR4 and PAI1 pathways in this regenerative phenotype and that at least one, if not both pathways/mechanisms are required for PRG4 mediated auricular cartilage regeneration (maturation). To further elucidate these potential mechanisms, we focused on the role of rhPRG4 in chondrogenesis as there appeared to be two parallel regenerative mechanisms in play, wound closure, and auricular cartilage formation.

### rhPRG4 induces progenitor cells to undergo chondrogenesis in vitro and in vivo

**Fig. 6:**
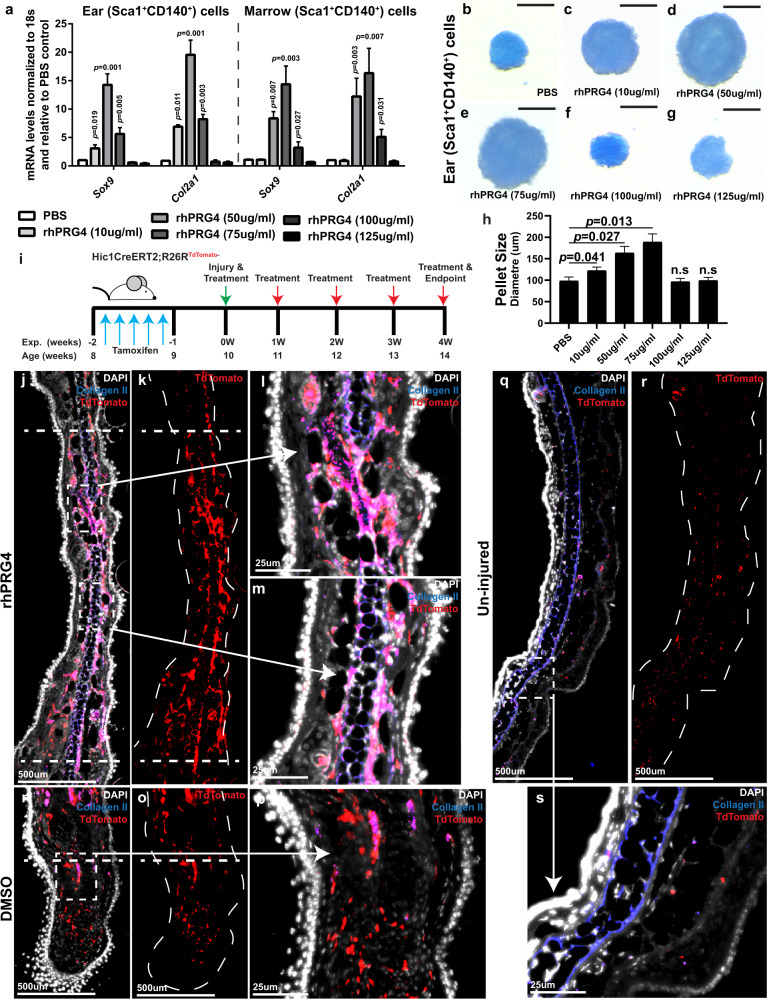
Effects of rhPRG4 on chondrogenesis in vitro and in vivo. Sca1^+^CD140a^+^ cells were isolated from the ear and bone marrow, exposed to increasing concentrations of rhPRG4 and assayed by qPCR **a** and alican blue staining **b**–**g**. Ear derived progenitors demonstrated a maximal beneficial chondrogenic effect at 50 µg/ml rhPRG4 in both marker expression **a** and pellet size **b**–**h**, while marrow derived progenitors demonstrated a maximal beneficial chondrogenic effect at 75 µg/ml rhPRG4 **a**. *Hic1*^+^ progenitors were permanently labeled in vivo with TdTomato by Tamoxifen induction pre-injury and their localization was examined 4 weeks post-injury **h** in presence/absence of rhPRG4 **i**–**o** (*n* = 6 mice per group). With rhPRG4 treatment, *Hic1*^+^ progenitors had given rise to auricular cartilage (overlap of TdTomato (red) with Collagen II (blue)) **i**–**l**, while in DMSO treated injuries, on minimal TdTomato^+^ signal was observed co-localized with Collagen II^+^ signal and this staining was not morphological consistent with auricular cartilage **m**–**o**. In uninjured ear tissue, few TdTomato^+^ cells were observed dispersed throughout the dermis and adjacent to the auricular cartilage. Few to no TdTomato^+^ Collagen II^+^ cells were observed **p**–**r**. In vitro experiments were undertaken on 3 biological and 3 technical replicates, in vivo experiments were undertaken on *n* = 4 animals per group. Bars represent mean with SD. n.s. = not significant. Error bars equal mean ± SD (**a**, **h** – 1 way ANNOVA).

While PRG4 is known to be essential for cartilage homeostasis^19,30,33^ and that osteochondral progenitor cells in the joint environment express *Prg4*^34,35^, little is known regarding the role of *Prg4* in chondrogenesis. Therefore, we isolated presumptive MPCs (Sca1^+^CD140a^+^) from the ear and (bone) marrow and induced these cells to undergo chondrogenic differentiation in the presence of rhPRG4 (Fig. 6a). While increasing concentrations of rhPRG4 led to increased expression of chondrogenic markers, a plateau was observed in the response of both ear and bone marrow MPCs. Chondrogenic gene expression peaked with 50 µg/mL rhPRG4 in ear MPCs and at 75 µg/mL in marrow MPCs (Fig. 6a). Chondrogenic pellet size followed the same trend as gene expression (Fig. 6b–h). To determine if rhPRG4 induced chondrogenic differentiation of MPCs in vivo, *Hic1*^CreERT2^:Rosa^tdTomato^ reporter mice^36,37^ were induced with tamoxifen to fate map *Hic1*^+^ MPCs and their progeny in response to full-thickness ear injury with/out rhPRG4 treatment (Fig. 6i). In mice treated with rhPRG4, enhanced wound closure was observed including the regeneration of the auricular cartilage as shown by the Collagen II staining (Fig. 6j–m). *De novo* auricular cartilage tissue was tdTomato^+^ Collagen II^+^, demonstrating that it was derived from *Hic1*-lineage cells (Fig. 6l, m). In mice treated with carrier alone (DMSO), little wound closure was observed, and minimal *do novo* auricular cartilage was seen within the injury site (Fig. 6n–p). Few tdTomato^+^ cells were co-localized with Collagen II^+^ staining, yet this tissue was not consistent with mature auricular cartilage (Fig. 6p). Flow cytometry was used to quantify the number of tdTomato^+^Collagen II^+^ double positive cells within the wound area and it was found that injuries treated with rhPRG4 demonstrated a significant increase in this population (Supplementary Fig. 11). In uninjured ear tissue, little to no co-localization was observed between tdTomato^+^ and Collagen II^+^ staining (Fig. 6q–s).

### MPCs upregulate Prg4 in response to injury while macrophages respond by increasing Vegf

**Fig. 7:**
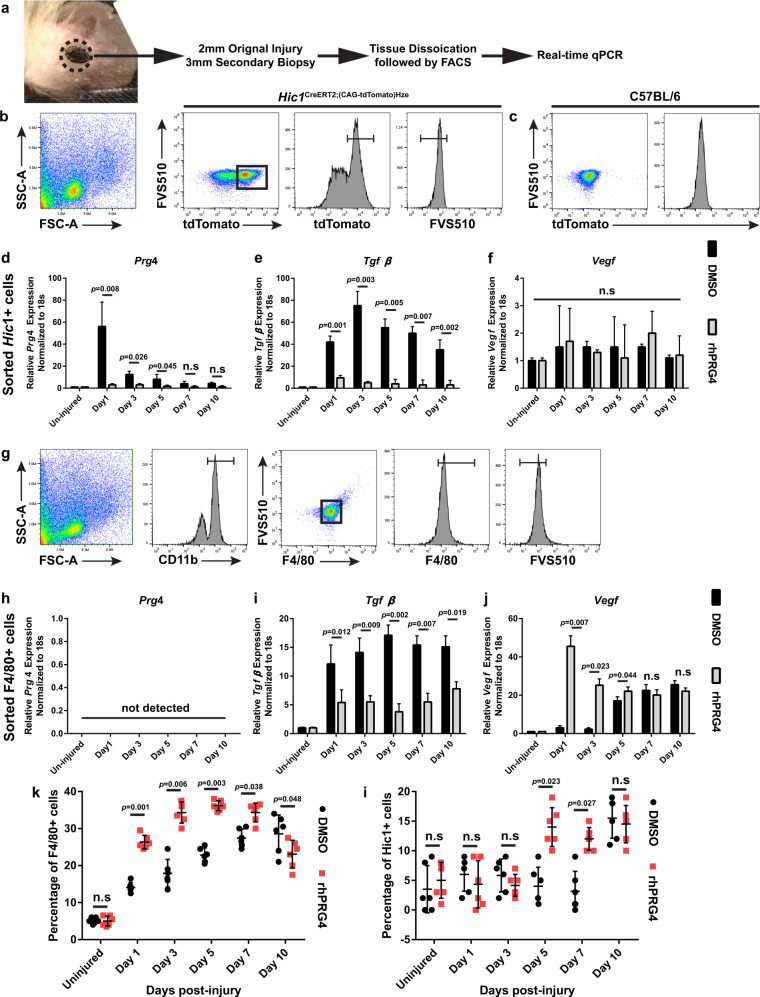
Gene expression in isolated progenitors and macrophages with rhPRG4 treatment. A 3 mm secondary biopsy was generated around the primary 2 mm wound injury at various timepoints post-injury and the isolated cells were purified and examined by qPCR **a**. Live, tdTomato^+^ **b**, **c**, *Hic1*^+^ progenitors were assayed for *Prg4*, *Tgfβ* and *Vegf* expression **d**–**f**. Both *Prg4* and *Tgfβ* levels were upregulated by rhPRG4, while no *Vegf* response was observed to rhPRG4. Live, CD11b^+^, F4/80^+^ macrophages **g** were assayed for *Prg4*, *Tgfβ* and *Vegf* expression **h**–**j**. *Prg4* was not detected in macrophages, however both *Tgfβ* and *Vegf* levels were upregulated by rhPRG4. The number of *Hic1*^+^ and F4/80^+^ cells were quantified post-injury and it was observed that F4/80^+^ macrophage numbers increased immediately after rhPRG4 treatment **k**; while *Hic1*^+^ cells increased at day 5 post-rhPRG4 treatment/injury **l**. All experiments were undertaken on at least 3 biological and 3 technical replicates unless otherwise stated. n.s. = not significant. Error bars equal mean ± SD (**d**, **e**, **f**, **h**, **I**, **j**, **k**, **l** – t-test).

To determine if and how MPCs respond to rhPRG4 post-injury, mice underwent ear injury followed by injury site biopsy at different endpoints. Live (FVS510^−^), tdTomato^+^ (*Hic1*^+^) MPCs were isolated (Fig. 7a, b, Supplementary Fig. 12) with tdTomato signal being determined based on cells from a tdTomato^−^ mouse (Fig. 7c). qPCR analysis on isolated cells revealed that in the absence of rhPRG4, *Hic1*^+^ MPCs upregulate *Prg4* expression at 1 day post-injury, with the levels gradually decreasing until day 7 post-injury, at which time no difference in expression was observed relative to the uninjured state (Fig. 7d). Interestingly, this effect is mitigated with the addition of rhPRG4 (Fig. 7d), demonstrating a feedback mechanism in this pathway. The same trend was observed with *Tgfβ* expression (Fig. 7e), demonstrating that exogenous rhPRG4 inhibits *Tgfβ* expression in *Hic1*^+^ MPCs post-injury. Since our earlier data showed that VEGF is upregulated in response to rhPRG4 treatment, we assayed *Hic1*^+^ MPCs for *Vegf* expression, but found no change in *Vegf* levels post-injury with/without rhPRG4 treatment (Fig. 7e). Since rhPRG4 inhibited *Tgfβ* expression, we also tested if this interaction played a role in contraction (Supplementary Fig. 13). There were significant increases in collagen gel contraction with rhPRG4, rhTGFβ or the combination of both, but no differences were observed between treatment groups. The TGFβ receptor inhibitor SB431542 blocked rhTGFβ induced contraction with/without rhPRG4 (Supplementary Fig. 13), further demonstrating an interaction of PRG4 and TGFβ pathways.

To determine if rhPRG4 was also regulating the behaviour of macrophage populations, live (FVS510^−^) macrophages (CD11b^+^F4/80^+^) were isolated from the normal and injured ears (Fig. 7g, Supplementary Fig. 12) and assayed by qPCR. Unlike, *Hic1*^+^ MPCs, no *Prg4* expression was observed in macrophages with/without rhPRG4 (Fig. 7h). Similar to *Hic1*^+^ MPCs, *Tgfβ* expression was increased post-injury and was muted in the presence of rhPRG4 (Fig. 7i). Interestingly, it appeared that macrophages upregulated expression of *Vegf* in response to injury, and this was enhanced in the presence of rhPRG4 with *Vegf* expression, gradually decreasing over time, reaching carrier alone levels by day 7 post-injury (Fig. 7j).

To determine if rhPRG4 influenced the numbers of macrophages and MPCs in the injury site, these cell types were quantified. rhPRG4 treatment resulted in a significant increase in F4/80^+^ macrophages within the wound site at 1 day post-injury and this persisted until 10 days post-injury, at which time there were more macrophages present in the vehicle treated wounds (Fig. 7k). There was no difference in MPC numbers until day 5 post-injury at which point more MPCs were present with rhPRG4 treatment (Fig. 7l). However, by day 10 post-injury the number of MPCs in carrier alone group (DMSO) reached the same level as rhPRG4 treated injuries (Fig. 7l). To further investigate this observation, the number of proliferating cells and proliferating MPCs within the injury site were investigated by flow cytometry analysis (Supplementary Fig. 14). The number of Ki67^+^ cells increased with rhPRG4 treatment and this was maintained over the first 10 days post-injury (Supplementary Fig. 14a, c). When the proportion of the Ki67^+^ population expressing MPC markers (Sca1^+^CD140a^+^) was examined, a dramatic increase in the number of proliferating progenitors was observed at 1 day post-injury and was maintained until day 5 post-injury (Supplementary Fig. 14b, d). However, by 10 days post-injury, this trend was reversed in where the number of proliferating MPCs in C57BL/6 mice treated with DMSO was significantly increased vs. mice treated with rhPRG4 (Supplementary Fig. 14b, d).

### Macrophages upregulate Hif1α and Vegf through the NFκB pathway

Since rhPRG4 treatment upregulated *Vegf* expression in macrophages, we examined this mechanism in more detail. We designed an NFκB reporter plasmid which expressed tdTomato when NFκB is activated (Supplementary Fig. 15a) and transfected it into primary monocytes, followed by differentiation into macrophages. Macrophages treated with/without rhPRG4 were assayed using flow cytometry and gated on the GFP positive cells (expressed in all cells with the plasmid). In the absence of rhPRG4, minimal tdTomato expression was detected, however, exposure to rhPRG4 induced tdTomato expression indicating activation of the NFκB pathway in nearly all GFP-expressing cells (Supplementary Fig. 15b, c). When these cells were assayed for *Hif1α* and *Vegf* expression, both were increased in the presence of rhPRG4 (Supplementary Fig. 15d). To determine if this increase was NFκB dependent, cells were treated with 30 µM JSH 23 (NFκB translocation inhibitor) and both *Hif1α* and *Vegf* expression were inhibited suggesting that their increase in macrophages is NFκB dependent (Supplementary Fig. 15d).

The same approach was undertaken in MPCs (Sca1^+^CD140a^+^), and while we observed NFκB activation in these cells with rhPRG4 treatment (Supplementary Fig. 15b, c), here were no increases in *Hif1α* or *Vegf* expression (Supplementary Fig. 15d), which was consistent with our in vivo data (Supplementary Fig. 15f).

### rhPRG4 regulates macrophage polarization state through TLR4

**Fig. 8:**
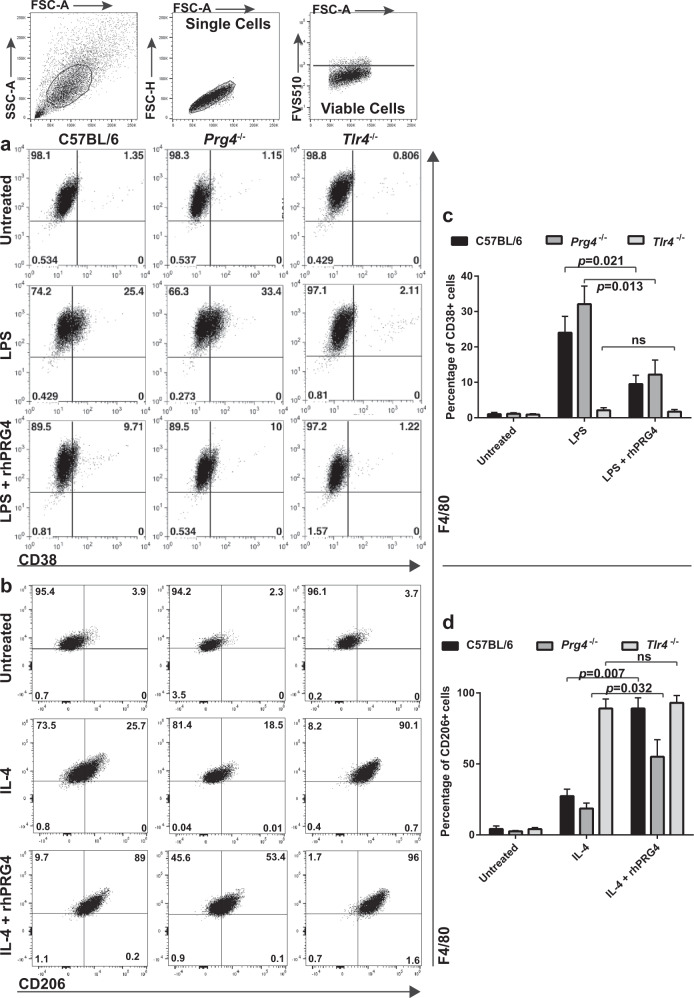
rhPR4G4 treatment of macrophages inhibits pro-inflammatory and enhances anti-inflammatory polarization. Bone marrow derived macrophages from C57BL/6, *Prg4*^−*/*−^ and *Tlr4*^−*/*−^ mice were stimulated with LPS or IL-4 to induce pro(CD38^+^)/anti(CD206^+^)-inflammatory polarization respectively. rhPRG4 was supplemented to LPS or IL-4 conditions to observe the effects of exogenous PRG4 on CD38^+^/CD206^+^ macrophages. Both C57BL/6 and *Prg4*^−*/*−^ macrophages were sensitive to CD38^+^ polarization by LPS, while *Tlr4*^−*/*−^ macrophages were not. While supplementing rhPRG4 in LPS conditions had no effects on *Tlr4*^−*/*−^ macrophage CD38^+^polarization; rhPRG4 reduced CD38^+^ polarization in C57BL/6 and *Prg4*^−*/*−^ macrophages. All macrophages were responsive to IL-4 polarization to CD206^+^ macrophages, however, *Tlr4*^−*/*−^ macrophages demonstrated robust CD206^+^ polarization. C57BL/6 and *Prg4*^−*/*−^ macrophages demonstrated an increased CD206^+^ polarization in the presence of rhPRG4, while it had no effect on *Tlr4*^−*/*−^ macrophage CD206^+^ polarization. All experimental were undertaken on at least 3 biological and 3 technical replicates unless otherwise stated. Error bars equal mean ± SD (**c**, **d**, – t-test). n.s. = not significant.

Since rhPRG4 regulated gene expression in macrophages, we sought to determine if rhPRG4 was able to modify macrophage functional state (Fig. 8). Monocytes were isolated from C57BL/6, *Prg4*^−/−^ and *Tlr4*^−/−^ mice and differentiated to macrophages, then polarized with LPS or IL4. Unstimulated macrophages from all mice examined showed no spontaneous expression of CD38. Exposure to LPS induced expression of CD38 in C57BL/6 and *Prg4*^−/−^ mice, but not in *Tlr4*^−/−^ mice. Furthermore, the number of cells adopting a CD38^+^ phenotype was elevated in *Prg4*^−/−^ relative to C57BL/6 mice (Fig. 8a, c). When LPS was supplemented with rhPRG4, the number of macrophages expressing CD38 was reduced in *Prg4*^−/−^ and C57BL/6 mice (Fig. 8a, c).

When examining the polarization of macrophages to an anti-inflammatory phenotype, in the absence of IL4, spontaneous expression of CD206 was observed. While macrophages from all three mice examined increased CD206 expression in response to IL4, *Tlr4*^−/−^ mice showed a particularly potent response with nearly 100% of cells expressing CD206 compared to approx. 25% of the cells in C57BL/6 and *Prg4*^−/−^ mice (Fig. 8b, d). When IL4 was supplemented with rhPRG4, the number of macrophages expressing CD206 was increased in *Prg4*^−/−^ and C57BL/6 mice, with C57BL/6 reaching CD206^+^ frequencies similar to *Tlr4*^−/−^ mice. Yet, even with rhPRG4 treatment, CD206 levels in *Prg4*^−/−^ were lower than C57BL/6 and *Tlr4*^−/−^ mice (Fig. 8b, d).

## Discussion

It is becoming increasingly clear that the interactions between immune cells and mesenchymal progenitor cells (MPCs) during wound healing are important determinants of healing outcomes^38^. The results of our current study suggests that PRG4 is a potent determinant of wound healing outcomes and may impact the behaviour of immune and MPC populations, biasing the wound healing response towards regeneration vs fibrotic repair.

PRG4/lubricin/superficial zone protein (SZP)/megakaryocyte-stimulating factor (MSF) was originally identified 40 years ago^15,39^ and has primarily been studied as a lubricant molecule essential for the health and homeostasis of articular cartilage. However, over the past decade, PRG4 has been found to play roles in regulating inflammation (at the cytokine and immune cell levels)^19,40–42^ and fibrosis^22^. It is becoming clear that PRG4 is involved in a number of biological processes in addition to lubrication and that the loss/absence of this protein can lead to a loss of normal homeostasis in multiple tissues/organ systems^40,43^. In this study, we demonstrate that the absence of PRG4 results in the severe impairment of wound closure in the ear punch model, with wounds showing little to no repair, even getting larger over time. This clearly shows that endogenous levels of PRG4 play an important role in the wound healing response and this is in agreement with a recent study demonstrating that BMP9 induced digit tip regeneration is lost in *Prg4*^−/−^ mice^21^. Within the current study, we have been able to show that PRG4 influences a number of pathways that have been previously implicated in the wound healing cascade (e.g. inflammation, proliferation and remodelling).

In terms of regulating inflammation, we demonstrate that PRG4 can modulate the polarization of macrophages and the expression of *Vegf* through TLR dependent mechanisms. Interestingly, the ability of PRG4 to induce *Vegf* through TLR4 appears to be Myd88 independent, while the polarization of macrophages to a CD38^+^ phenotype in *Prg4*^−*/*−^ mice is enhanced by treatment of LPS which functions through a Myd88 dependent pathway. This is consistent with prior research demonstrating that TLR4 signalling through both MyD88 and TRIF is important in the maturation of macrophages and dendritic cells^44^. Furthermore, we have previously demonstrated that PRG4 can induce NFκB nuclear translocation through TLR2,4,5 and 9 and while we focused on TLR4 in the current study, it is possible that even in the absence of TLR4 (*Tlr4*^−/−^), PRG4 could still be effecting changes in signalling through other TLRs, CD44^41^ or other receptors/pathways. To our knowledge, this is the first study demonstrating PRG4 treatment induces the expression of *Vegf* in vitro and can result in increased angiogenesis in vivo post-injury. We were also able to demonstrate that the *Vegf* was produced by macrophages and not MPCs (based on the markers employed). At first glance, this may seem counter intuitive as the highest concentration of PRG4 in the body is found on the articular cartilage surface, yet the cartilage is not vascularized. Furthermore, it has been demonstrated the majority of chondrocytes in normal and arthritic cartilage do express *Vegf* (transcript and protein)^45^. This implies that angiogenesis in cartilage is inhibited^46^ downstream of VEGF and therefore does not conflict with our observation that PRG4 induces expression of VEGF. There is evidence in the literature that macrophages are potent drivers of the pro- angiogenic response^47,48^ and that this effect is dependent on NFκB signalling^49^. This is consistent with our findings that PRG4 induced the expression of *Vegf* in an NFκB dependent manner in macrophages. Yet, it should be noted that while PRG4 also induced NFκB signalling in MPCs, this did not result in the upregulation of *Vegf* (nor was this observed in vivo), which was surprising since MPCs are also known to produce *Vegf*^50,51^. However, there is research demonstrating that PRG4 can influence the behaviour of macrophages^52–54^ and neutrophils^55^ and therefore it is possible that the immunomodulatory and pro-angiogenic effects of PRG4 are biased towards immune cell populations, yet this remains a hypothesis and would need to be directly tested, potentially through macrophage depletion and/or conditional deletion of *Prg4* in macrophage subsets. Our results may also be specific to the MPC populations tested and cells derived from other tissue sources may exhibit a differential VEGF post-PRG4 treatment effect.

In addition to regulating VEGF expression, we observed that the level of PRG4 modified macrophage phenotype (pro vs. anti-inflammatory) through a TLR4-dependent mechanism which is consistent with a recently published study^54^. Specifically, the absence of PRG4 biases macrophage populations to a pro-inflammatory phenotype, while the addition of exogenous PRG4 biases macrophages to an anti-inflammatory phenotype. Yet, in *Tlr4*^−/−^ mice, the addition of exogenous PRG4 had no effect on macrophage phenotype. This strongly suggests that the ability of PRG4 to regulate macrophage phenotype is regulated primarily through TLR4. This is consistent with the literature showing that the pro-inflammatory phenotype is TLR4-dependent^56,57^ and that the absence of TLR4 expression biases macrophages to the anti-inflammatory phenotype^58^. We have previously shown that PRG4 binding to TLR4 can partially inhibit downstream signalling^19^ and therefore, induce a similar outcome to that seen in *Tlr4*^−/−^ mice. Our current results show that *Tlr4*^−/−^ mice demonstrated enhanced wound healing, partly through regulation of VEGF, but also likely through modification of macrophage phenotype. However, this observation is not consistent with previous findings demonstrating that *Tlr4*^−/−^ mice demonstrate decreased wound healing ability^59,60^. This could be due to the differences in the injury induced (skin vs. ear) and therefore, it would be interesting in future work to determine if PRG4 treatment could influence full thickness skin injuries and if TLR4 had any impact on this effect. We also observed a significant increase in the number of proliferating cells and specifically MPCs in the wound post-rhPRG4 treatment suggesting that PRG4 is also able to regulate the cell cycle. This role is backed up the literature which demonstrates that *Prg4*^−*/*−^ mice display spontaneous synovial overgrowth^22,42^. We would also like to note that we saw significantly more proliferative cells in the wound area than previous studies^12^, but we believe this is likely due to our sampling method in where we only re-punched the 2 mm wound with a 3 mm punch to obtain tissue for these assays. This methodology is biased towards cell populations present on the edge of the wound margin.

Within the multiple transgenic lines used to investigate regeneration in this study, it was interesting to observed that the most potent cartilage repair/regeneration was observed in the presence of PRG4, even in mouse lines which demonstrated increased endogenous wound closure. This result was validated in vitro, where it was shown that PRG4 induced chondrogenesis is a dose-dependent manor. To our knowledge, this is the first time that PRG4 has been shown to induce the chondrogenesis, despite that fact that it is commonly used as a marker of chondrocytes^19,34,61,62^. However, there have been at least three independent studies suggesting that the loss of PRG4 increases chondrogenesis^33,63,64^. While our results may appear to conflict with the published record, based on our current results, we suggest that the PRG4 dose and the cell type it is acting upon could greatly influence the outcome. Specifically, we observed distinct dose-response kinetics between ear vs. bone marrow MPCs with higher concentrations of PRG4 inhibiting chondrogenesis. We further hypothesize that this observation may be in part dependent on TGFβ, as this growth factor is a potent driver of the chondrogenic program^65,66^. Our results clearly demonstrate that within MPCs, the addition of PRG4 inhibits the expression of *Tgfβ*, this is an important and novel finding since it is known that TGFβ drives the expression of *Prg4*^63,67,68^. Therefore, at specific doses, there might be synergy between TGFβ and PRG4, while at higher doses of PRG4, TGFβ signalling and by extension, chondrogenesis, maybe be abrogated. If this hypothesis is correct, then this could explain the incomplete regeneration/neo-chondrogenesis observed in *Prg4*^−/−^ mice post-PRG4 treatment. Furthermore, since both the PAI1 and TLR4 pathways regulate and are regulated by TGFβ^69–73^, this could also explain the lack of neo-chondrogenesis in *Pai1*^−/−^ and *Tlr4*^−/−^ mice treated with PRG4. The notable exception was the *Pai1*^−/−^*Tlr4*^−/−^ mice treated with PRG4, which demonstrated robust neo-chondrogenesis in the wound area. If inhibiting both PAI1 and TLR4 pathways reset TGFβ to a default level, then this would explain this result. However, this result could also be independent of TGFβ signalling, and therefore additional in vitro and in vivo experiments would be required to further characterize the mechanism(s) involved.

Overall, we have demonstrated that pathways regulated by PRG4 in the wound healing response bias regeneration vs. repair and this finding has the potential to improve healing following connective tissue injury.

## Methods

### Study design

The objective of this study was to evaluate the therapeutic potential of rhPRG4 in treating full thickness ear wound injuries in mice. For in vivo experiments, 10-week-old mice were used, with equal numbers of males and females in each group. Animal size sample was determined by power analysis based on preliminary data. Mice were randomized across treatment groups. This study was carried out in accordance with the recommendations in the Canadian Council on Animal Care Guidelines. Animal protocols and surgical procedures were approved by the University of Calgary Animal Care Committee (protocol AC16-0043 and AC20-0042). All surgery was performed under isoflurane anaesthesia. Number of repeats is specified in each figure legend.

### Animals

C57Bl/6 J; *Prg4*^−/−^ (*Prg4*^*tm1Mawa*^); *Tlr4*^−/−^ (*Tlr4*^*tm1.2Karp*^/J); *Pai1*^−/−^ (*Serpine1*^*tm1Mlg*^) mice were purchased from Jackson Laboratories (Bar Harbor, Maine). *Prg4*^−/−^ were backcrossed to C57BL/6 J for 5 generation, then genotyped to confirmed absence of *Prg4* before use. *Hic1*^CreERT2^:Rosa^tdTomato^ mice (referred to as “*Hic1*”) were obtained from the Underhill lab at the University of British Columbia. All animals were fed a standard diet and housed under a standard light cycle. Equal numbers of male and female mice were used and equally distributed across each treatment/control group unless otherwise stated.

### In vivo ear punch wound healing model

Ten week old mice were used for the ear wound healing experiments. All mice were placed under isoflurane anaesthesia and a 2 mm through and through ear punch was given to the centre of the left ear at week 0. Mice were assigned to one of several treatment groups. Treatments were applied once per week and mice were sacrificed at 7, 14, 21 or 28 days post-injury. Images of the wound were captured each week or 4 weeks with a size standard included in each frame. Images were analyzed for wound diameter using ImageJ software.

### Recombinant PRG4 preparation and treatment

Full length recombinant human lubricin protein (rhPRG4) was derived from Chinese Hamster Ovary cells (Lubris, LLC). Briefly, the gene encoding the full length 1404 amino acid human PRG4 was inserted into plasmid vectors (Selexis SA), then the protein was subjected to ultrafiltration/diafiltration, a 3-step chromatographic purification process^19,74^. rhPRG4 in DMSO (100 µg/mL rhPRG4 in 10 µl DMSO) or DMSO alone was topically applied to the injury site once per week using a Contec™ CONSTIX™ lint-free, non-absorbent applicator (Fisher). The solution was applied in a circular motion at the inner edge of the injury site.

### Lubrication test

A Bose ELF 3200 (BOSE ElectroForce Systems Group) was used to analyze the boundary lubrication ability of PBS, DMSO, rhPRG4, rhPRG4 + DMSO and human synovial fluid in a cartilage-glass testing setup^75^. In each test setup all samples were compressed by 18% of the total cartilage thickness. Samples were allowed to stress‐relax for 40 min to enable fluid depressurization of the interstitial fluid. Samples were then rotated ±2 revolutions at varying effective sliding velocities (*v*_eff_ = ω · *R*_eff_, where *R*_eff_ is effective radius (=2.4 mm) and ω is angular velocity) of 0.3 mm/s; with a pre‐sliding duration of 120 s between rotation.

### In vivo blood flow analysis

Angiogenesis and blood flow analysis was measured by laser speckle imaging (Omegawave Inc, Japan) as done previously^76^. With the mouse under anesthesia, the imaging camera was focused such that the laser beam illuminated the entire dorsal surface of the ear. Perfusion points were color-coded for immediate visual display of blood flow maps. A series of 10 blood flow images were saved at 1 s intervals and analyzed to produce average perfusion values within a donut shaped analysis region around the wound.

### Histology

After sacrifice, ears were harvested and fixed in neutral buffered formalin (NBF) (Sigma) for 48 h. Samples then underwent tissue processing using a Leica TP1020 automatic tissue processing machine. Tissue was then embedded in paraffin wax blocks (all in the same orientation) and sections were cut with Leica Automated Rotary Microtome (RM 2255) at 8 µm sections and transferred onto slides. The mid-point of each injury was used for analysis (represented by the dashed black bar in the Figures), and the original margins of the injury was demonstrated by the parallel black bars. Slides were imaged using a Plan-Apochromat objective (10x/0.8 M27) on an Axio Scan.Z1 Slide Scanner microscope (Carl Zeiss). Images were compiled and analyzed with Zeiss Zen software.

### Immunofluorescence

Paraffin embedded sections (10 µm) were deparaffinized in CitriSolv (Fisher Scientific; Fairlawn, NJ) and rehydrated through a series of graded ethanol to distilled water steps. Antigen retrieval (10 mM sodium citrate, pH 6.0) and blocking (1:500 dilution; 100 µL rat serum:50 mL TRIS-buffered saline, 0.1% Tween 20 (TBST) for 1 h) was undertaken prior to sequential washes (TBST) and primary antibody incubation. Primary antibodies (VEGF [clone # VG1 – Invitrogen Cat# MA5-12184, used at 1:100], CD31 [clone #390 – Invitrogen Cat# 14-0311-82, used at 1:100], Col2 [clone # II-II6B3 – DSHB, Iowa, used at 1:50], αSMA [clone # 1A4 – ABCAM Cat# ab7817, used at 1:100] and PRG4 [clone # 9G3 – Millipore, Cat# MABT401, used at 1:100]) were directly conjugated to fluorophores using the DyLight® 488, 550 or 650 Conjugation Kit [ABCAM]. All slides were mounted using EverBrite™ Hardset Mounting Medium with DAPI (Biotium) for nuclear counterstaining and coverslipped. Slides were imaged using a Plan-Apochromat objective (10x/0.8 M27) on an Axio Scan.Z1 Slide Scanner microscope (Carl Zeiss); DAPI (353 nm/465 nm), EGFP/FITC/Alexa Fluor 488 (493 nm/517 nm), R-PE (565 nm/576 nm), and APC (650 nm/660 nm) specific filters were employed. Isotype controls for Alexa Fluor 488, 550 or 650 demonstrated little to no reactivity.

### Western blot analysis

Total protein from cultured cells or cells isolated directly from tissue was collected using a Tris-HCl/SDS based lysis/sample buffer and separated on a 10% poly-acrylamide gel. The gels were transferred to nitrocellulose membranes and probed with primary antibodies specific to the proteins VEGF and Histone H3 (Rabbit polyclonal – Invitrogen Cat#PA5-16183, used at 1:1000). Histone H3 was utilized as a control, since it is constitutively expressed in high levels in most cell types. An infra-red (either Alexa Fluor 680 or 800) secondary was utilized for detection of the signal with the Odyssey imaging system (LICOR).

### Luminex assay

After mice were sacrificed, blood was collected from mice via cardiac puncture. Blood was allowed to form a clot before centrifugation and collection of serum. Total protein from cultured from tissue was collected using a Tris-HCl/SDS based lysis/sample buffer. Serum and tissue homogenate was assayed using the Luminex platform (Eve Technologies, Calgary, AB, Canada). VEGF levels were quantified using a mouse custom discovery assay (Millipore) and prepared standards were included in all runs. All samples were assayed in duplicate.

### Transgenic TLR cell assays

TLR Null, TLR Null-2, TLR-2, −4, and −5 cell lines (Invivogen) were exposed to either positive controls for the TLRs (Heat-killed Listeria Monocytogenes:HKLM for TLR-2 (108 cells/mL), LPS for TLR-4 (100 ng/mL), and FLA for TLR-5 (100 ng/mL)) or rhPRG4 (100 µg/mL) in the presence of absence of the MyD88 inhibitor ST2835 (10 µM, Cedarlane). The cells and ligands were then plated and incubated at 37 °C, 5% CO2 over a 24-h period in HEK Blue media (Invivogen) then assayed at 630 nm.

### Surface Plasmon Resonance (SPR)

Binding of rhPRG4 to PAI1 was assessed using a Biacore X100 SPR instrument (GE Healthcare). Human PAI1 (Peptrotech) was immobilized onto the flow cell 2 of CM5 sensor chip (GE Healthcare) using standard amine-coupling chemistry, resulting in 300–500 response units (RU) The reference cell (flow cell 1) was prepared by activation and deactivation. The binding assay was performed in PBS running buffer supplemented with 0.01% (v/v) Tween 20. rhPRG4 solution was buffer exchanged to running buffer and at least 5 concentrations in the range of 2.74–2000µg/mL were injected at a flow rate of 30 µL/min with a contact time of 1 min at 25 °C. The bound lubricin was removed from the chip surface by injecting 1 M NaCl after monitoring dissociation for 1.5 min.

### Ear tissue dissociation

In experiments where RNA was collected and/or single cells were required for cell sorting/flow cytometry; the ear tissue surrounding the injury area was removed using a 3 mm biopsy punch and the resultant tissue was dissociated using the Multi Tissue Dissociation Kit 1 (Miltenyi Biotec), following the Dissociation of mouse ear following the Multi Tissue Dissociation Kit 1 protocol.

### Fluorescent activated cell sorting (FACS)

Cells isolated from ear tissue or bone marrow were washed with DPBS, stained with known MPC or macrophage markers for 30 minutes on ice in the dark, then washed again and placed into culture media in preparation for cell sorting. The MPC markers included were: Sca-1 (clone D7, Cat#14-5981-82, used at 1 µl/1million cells), CD140 (clone APA5, Cat#14-1401-82, used at 1 µl/million cells), both ThermoFisher. A cell viability marker was also employed, fixable viability stain (FVS) 510 (BV510) (BD Biosciences). UltraComp eBeads (eBioscience) individually stained with each single colour as well as unstained cells were used as compensation controls for the experiment.

The stained cells underwent fluorescent activated cell sorting (FACS) using a cell sorter (BD FACS Aria Fusion (BD Biosciences). For MPC isolation, viable (FVS510^−^) Sca1^+^CD140a^+^ double positive cells were isolated from bone marrow and ear tissue. For *Hic1*^+^ MPCs, viable (FVS510^−^) Tdtomato^+^ positive cells were isolated from ear tissue. For macrophage isolation, viable (FVS510^−^) CD11b^+^F4/80^+^ (both ThermoFisher – CD11b, clone # M1/70, Cat# 17-0112-82, used at 1 µl/1million cells; F4/80, clone # BM8, Cat# MF48000, used at 1 µl/1million cells) double positive cells were isolated from ear tissue. *Hic1*^+^ and CD11b^+^F4/80^+^ double positive cells were directly transferred to TRIzol for RNA isolation and qPCR analysis, while Sca1^+^CD140a^+^ double positive cells were sorted into cell culture media containing DMEM/F-12 media (Gibco) with 10% MSC stimulatory supplement (Stem Cell Technologies mesenchymal stem cells stimulatory supplements: mouse) with 1% antibiotic-antimycotic (ThermoFisher) until a sufficient number of MPCs were obtained for chondrogenesis.

### 3-D collagen gel contraction assay

The 3-D collagen gel contraction assay was performed as previously described. Medium containing 10 ng/mL human recombinant TGF-β1 (with/without SB431542, Sigma) or PAI1 was added into each well, with or without 100 μg/mL rhPRG4. Images of the collagen matrix gel surface area were obtained 24 h from the time of release from the side of the wells. Percent contraction was measured using ImageJ.

### Ear tissue and monolayer cell culture quantitative PCR

RNA from dissociated mouse ear tissue sorted (*Hic1*^+^, Sca1^+^Cd140a^+^ or CD11b^+^F4/80^+^ cells) or monolayer cultured cells (TLR Null, TLR Null-2, TLR-2, −4, and −5 cell lines) was extracted using E.Z.N.A® Total RNA Kit I (OMEGA). RNA was converted to cDNA using High Capacity Reverse Transcriptase cDNA kit (Thermo-Fisher). mRNA levels were analyzed using TaqMan® Universal PCR Master Mix using TaqMan® Gene Expression Assay primers for mouse or human *Vegf*, *Hif1α*, *Tgf*β, *Prg4* and 18 S (endogenous control) on Quantstudio 6 Real-Time PCR System (all Thermo-Fisher). Samples were run in triplicate.

### Chondrogenesis

Mouse MPCs were pelleted at 50,000 cells per pellet in 1.5 mL Eppendorf tubes by centrifugation. Cells were cultured in chondrogenic media containing 500 ng/mL BMP-2 (Peprotech), 10 ng/mL TGF-β3 (Peprotech), 10 M dexamethasone, 50 μg/mL ascorbic acid, 40 μg/mL proline, 100 μg/mL pyruvate and supplemented with 1× insulin, transferrin and selenium (all Sigma), the pH of the final solution was adjusted to 7 with NaOH in addition to the respective rhPRG4 concentration for 21 days. Undifferentiated control was pelleted and cultured in media without any growth factors or rhPRG4. Media was changed every 2–3 days, with special attention not to disturb the pellet.

### qPCR for analysis of chondrogenesis

Pellets were lysed in TRIzol with a 21-gauge needle, then total RNA was extracted using E.Z.N.A® Total RNA Kit I (OMEGA). Briefly, 200 µL of chloroform per ml of TRIzol was added to the TRIzol solution. Samples were vortexed, incubated at room temperature for 15 minutes, then centrifuged for 15 minutes at 4 °C and 12000 rpm. The top, clear, layer was transferred into a new HiBind® RNA mini column in a 2 mL collection tube. The sample was centrifuged at 10,000 g for 1 min, and the filtrate was discarded. The sample was washed with 500 µL wash buffer I, centrifuged for 30 s at 10,000 g and the filtrate was discarded. The sample was washed with 500 µL of wash buffer II, centrifuged for 1 min at 10,000 g, and the filtrate was discarded. The sample was then centrifuged at 14,000 g for 2 min to remove any excess liquid. The collection tube was replaced with a new one and the sample was extracted by adding 50 µL of ultra-pure water (ThermoFisher) and centrifuging at 14,000 g for 2 min. The sample was immediately stored at −80 °C until the next step could be completed. RNA was converted to cDNA using a High Capacity Reverse Transcriptase cDNA kit, mRNA levels were then analyzed using TaqMan® Universal PCR Master Mix using TaqMan® Gene Expression Assay primers for mouse *Sox9*, Col2a1 and *18* *S* (endogenous control) (all ThermoFisher).

### Monocyte isolation and macrophage differentiation

Murine monocytes were isolated using the EasySep™ Mouse Monocyte Isolation Kit (StemCell Technologies) following the manufactures protocol. Isolated cells were incubated in DMEM supplemented with 10% heat-inactivated fetal bovine serum, Anti-Anti, and macrophage colony stimulating factor (all Thermo-Fisher). Cells were harvested 24 h post-stimulation and assayed by flow cytometry using antibodies towards F4/80 (pan-macrophage), CD38 (M1, ThermoFisher, clone # 90, Cat# 14-0381-82, used at 1 µl/1million cells) or CD204 (M2, ThermoFisher, clone # MR6F3, Cat# MA5-16871, used at 1 µl/1million cells).

### Reporter plasmid generation and lentiviral transduction

The NFκB reporter plasmid #49343 from Addgene (pHAGE NFkB-TA-LUC-UBC-GFP-W)^77^ was modified to create NFκB NLS-tdTomato, simply by replacing firefly luciferase with NLS-tdTomato. The plasmid was then packed into a lentiviral vector. Cells (monocytes or MPCs) were incubated with the following mix overnight (~12 h) at 37 °C, 2% O2: 5 mL respective culture medium, 5 µL lentivirus and 2uL of Polybrene (8 µg/mL, Sigma). Medium was changed the following day.

### Statistics

Statistics on mouse ear wound healing was done using a two-way ANOVA with a Bonferroni correction. This was used to determine significance relative to the treatment group relative to the control group. The p value cut off was set to 0.05. All other assays (RT-qPCR, western blot, blood flow among others) were analyzed with a standard one-way or two-way ANOVA (as appropriate) to determine the significance relative to the control. All statistical analysis were done using GraphPad Prism 6 software.

### Reporting summary

Further information on research design is available in the Nature Research Reporting Summary linked to this article.

## Supplementary information

Supplemental Figures

REPORTING SUMMARY

## Data Availability

All data are available in the main text or the supplementary materials.

## References

[1] Gurtner GC, Werner S, Barrandon Y, Longaker MT (2008). Wound repair and regeneration. Nature.

[2] Sen CK (2009). Human skin wounds: A major and snowballing threat to public health and the economy: PERSPECTIVE ARTICLE. Wound Repair Regeneration.

[3] Demidova-Rice TN, Hamblin MR, Herman IM (2012). Acute and impaired wound healing: pathophysiology and current methods for drug delivery, part 1: normal and chronic wounds: biology, causes, and approaches to care. Adv. Ski. Wound Care.

[4] Eming SA, Martin P, Tomic-Canic M (2014). Wound repair and regeneration: mechanisms, signaling, and translation. Sci. Transl. Med..

[5] Maxson S, Lopez EA, Yoo D, Danilkovitch-Miagkova A, Leroux MA (2012). Concise review: role of mesenchymal stem cells in wound repair. Stem Cells Transl. Med..

[6] Zhang Y (2015). Inhibition of the prostaglandin-degrading enzyme 15-PGDH potentiates tissue regeneration. Sci. (80).

[7] Bertram KL (2018). 17-DMAG regulates p21 expression to induce chondrogenesis in vitro and in vivo. Dis. Model. Mech..

[8] Seifert AW (2012). Skin shedding and tissue regeneration in African spiny mice (Acomys). Nature.

[9] Fitzgerald J (2008). Evidence for articular cartilage regeneration in MRL/MpJ mice. Osteoarthr. Cartil..

[10] Bedelbaeva K (2010). Lack of p21 expression links cell cycle control and appendage regeneration in mice. Proc. Natl Acad. Sci. USA.

[11] Clark LD, Clark RK, Heber-Katz E (1998). A new murine model for mammalian wound repair and regeneration. Clin. Immunol. Immunopathol..

[12] Gawriluk TR (2016). Comparative analysis of ear-hole closure identifies epimorphic regeneration as a discrete trait in mammals. Nat. Commun..

[13] Ikegawa S, Sano M, Koshizuka Y, Nakamura Y (2000). Isolation, characterization and mapping of the mouse and human PRG4 (proteoglycan 4) genes. Cytogenet. Cell Genet..

[14] Ai M (2015). Anti-lubricin monoclonal antibodies created using lubricin-knockout mice immunodetect lubricin in several species and in patients with healthy and diseased joints. PLoS ONE.

[15] Swann DA, Slayter HS, Silver FH (1981). The molecular structure of lubricating glycoprotein-I, the boundary lubricant for articular cartilage. J. Biol. Chem..

[16] Schmidt TA (2013). Transcription, translation, and function of lubricin, a boundary lubricant, at the ocular surface. JAMA Ophthalmol..

[17] Marcelino J (1999). CACP, encoding a secreted proteoglycan, is mutated in camptodactyly-arthropathy-coxa vara-pericarditis syndrome. Nat. Genet..

[18] Ciullini Mannurita S (2014). CACP syndrome: identification of five novel mutations and of the first case of UPD in the largest European cohort. Eur. J. Hum. Genet..

[19] Iqbal SM (2016). Lubricin/Proteoglycan 4 binds to and regulates the activity of Toll-Like Receptors In Vitro. Sci. Rep..

[20] Alquraini, A. et al. The interaction of lubricin/proteoglycan 4 (PRG4) with toll-like receptors 2 and 4: An anti-inflammatory role of PRG4 in synovial fluid. *Arthritis Res. Ther*. **17**, (2015). 10.1186/s13075-015-0877-x PMC4672561 26643105

[21] Yu, L. et al. BMP9 stimulates joint regeneration at digit amputation wounds in mice. *Nat. Commun*. **10**, (2019). 10.1038/s41467-018-08278-4 PMC6363752 30723209

[22] Qadri, M. et al. Proteoglycan-4 regulates fibroblast to myofibroblast transition and expression of fibrotic genes in the synovium. *Arthritis Res. Ther*. **22**, (2020). 10.1186/s13075-020-02207-x PMC7222325 32404156

[23] Mak, J. et al. Evaluating endogenous repair of focal cartilage defects in C57BL/6 and MRL/MpJ mice using 9.4T magnetic resonance imaging: A pilot study. *Magn. Reson. Imaging* **33**, (2015). 10.1016/j.mri.2015.01.001 25597446

[24] Jablonski, C. L., Besler, B. A., Ali, J. & Krawetz, R. J. p21−/− Mice exhibit spontaneous articular cartilage regeneration post-injury. *Cartilage* 1947603519876348 10.1177/1947603519876348 (2019). 10.1177/1947603519876348 PMC8804758 31556320

[25] Sari E, Bakar B, Dincel GC, Budak Yildiran FA (2017). Effects of DMSO on a rabbit ear hypertrophic scar model: a controlled randomized experimental study. J. Plast. Reconstr. Aesthet. Surg..

[26] Huang, S. et al. The level of synovial human VEGFA, IL-8 and MIP-1α correlate with truncation of lubricin glycans in osteoarthritis. bioRxiv 2021.03.11.434779 10.1101/2021.03.11.434779 (2021).

[27] Bhagwani, A., Thompson, A. A. R. & Farkas, L. When Innate Immunity Meets Angiogenesis—The Role of Toll-Like Receptors in Endothelial Cells and Pulmonary Hypertension. *Front. Med.* **7**, 352 (2020). 10.3389/fmed.2020.00352 PMC7410919 32850883

[28] Loiarro M (2007). Pivotal advance: inhibition of MyD88 dimerization and recruitment of IRAK1 and IRAK4 by a novel peptidomimetic compound. J. Leukoc. Biol..

[29] Emanuele, N. et al. Effect of recombinant lubricin on human blood coagulation parameters and platelet aggregation. *FASEB J*. **30**, lb458-lb458

[30] Rhee DK (2005). The secreted glycoprotein lubricin protects cartilage surfaces and inhibits synovial cell overgrowth. J. Clin. Invest..

[31] Jones, A. R. C. Functions of the structural domains of cartilage superficial zone proteoglycan/proteoglycan 4 (SZP/PRG4). -ORCA. PhD Thesis, Cardiff University http://orca.cf.ac.uk/55062/ (2004).

[32] Pincha N (2018). PAI1 mediates fibroblast-mast cell interactions in skin fibrosis. J. Clin. Invest.

[33] Abubacker S (2019). Absence of proteoglycan 4 (Prg4) leads to increased subchondral bone porosity which can be mitigated through intra-articular injection of PRG4. J. Orthop. Res..

[34] Kozhemyakina E (2015). Identification of a Prg4-expressing articular cartilage progenitor cell population in mice. Arthritis Rheumatol..

[35] Roelofs AJ (2020). Identification of the skeletal progenitor cells forming osteophytes in osteoarthritis. Ann. Rheum. Dis..

[36] Scott RW, Arostegui M, Schweitzer R, Rossi FMV, Underhill TM (2019). Hic1 defines quiescent mesenchymal progenitor subpopulations with distinct functions and fates in skeletal muscle regeneration. Cell Stem Cell.

[37] Abbasi S (2020). Distinct regulatory programs control the latent regenerative potential of dermal fibroblasts during wound healing. Cell Stem Cell.

[38] Rodrigues M, Kosaric N, Bonham CA, Gurtner GC (2019). Wound healing: a cellular perspective. Physiol. Rev..

[39] Swann DA, Sotman S, Dixon M, Brooks C (1977). The isolation and partial characterization of the major glycoprotein (LGP-I) from the articular lubricating fraction from bovine synovial fluid. Biochem. J..

[40] Das N, Schmidt TA, Krawetz RJ, Dufour A (2019). Proteoglycan 4: from mere lubricant to regulator of tissue homeostasis and inflammation. BioEssays.

[41] Al-Sharif A (2015). Lubricin/proteoglycan 4 binding to CD44 receptor: a mechanism of the suppression of proinflammatory cytokine-induced synoviocyte proliferation by lubricin. Arthritis Rheumatol..

[42] Alquraini A (2017). The autocrine role of proteoglycan-4 (PRG4) in modulating osteoarthritic synoviocyte proliferation and expression of matrix degrading enzymes. Arthritis Res. Ther..

[43] Melrose, J. A perspective on the potential utility of a viscosupplement multifunctional biotherapeutic: Proteoglycan-4: from mere lubricant to regulator of tissue homeostasis and inflammation. *Bioessays* **41**, (2019). 10.1002/bies.201800215 30500085

[44] Shen H, Tesar BM, Walker WE, Goldstein DR (2008). Dual signaling of MyD88 and TRIF is critical for maximal TLR4-induced dendritic cell maturation. J. Immunol..

[45] Pfander D (2001). Vascular endothelial growth factor in articular cartilage of healthy and osteoarthritic human knee joints. Ann. Rheum. Dis..

[46] Kern, B. E., Balcom IV, J. H., Antoniu, B. A., Warshaw, A. L. & Fernández-del Castillo, C. Troponin I peptide (Glu94-Leu123), a cartilage-derived angiogenesis inhibitor: In vitro and in vivo effects on human endothelial cells and on pancreatic cancer. in *J. Gastrointest. Surg.* **7** 961–969 (Elsevier Inc., 2003). 10.1016/j.gassur.2003.08.003 14675705

[47] Riazy M, Chen JH, Steinbrecher UP (2009). VEGF secretion by macrophages is stimulated by lipid and protein components of OxLDL via PI3-kinase and PKCζ activation and is independent of OxLDL uptake. Atherosclerosis.

[48] Wu WK, Llewellyn OPC, Bates DO, Nicholson LB, Dick AD (2010). IL-10 regulation of macrophage VEGF production is dependent on macrophage polarisation and hypoxia. Immunobiology.

[49] Kiriakidis S (2003). VEGF expression in human macrophages is NF-κB-dependent: Studies using adenoviruses expressing the endogenous NF-κB inhibitor IκBα and a kinase-defective form of the IκB kinase 2. J. Cell Sci..

[50] Ge Q (2018). VEGF secreted by Mesenchymal stem cells mediates the differentiation of endothelial progenitor cells into endothelial cells via paracrine mechanisms. Mol. Med. Rep..

[51] Chang YS (2014). Critical role of vascular endothelial growth factor secreted by mesenchymal stem cells in hyperoxic lung injury. Am. J. Respir. Cell Mol. Biol..

[52] Qadri M (2018). Recombinant human proteoglycan-4 reduces phagocytosis of urate crystals and downstream nuclear factor kappa B and inflammasome activation and production of cytokines and chemokines in human and murine macrophages. Arthritis Res. Ther..

[53] Nahon JE (2018). Proteoglycan 4 regulates macrophage function without altering atherosclerotic lesion formation in a murine bone marrow-specific deletion model. Atherosclerosis.

[54] Qadri, M. et al. Proteoglycan-4 is an essential regulator of synovial macrophage polarization and inflammatory macrophage joint infiltration. *Arthritis Res. Ther*. **23**, 241 (2021). 10.1186/s13075-021-02621-9 PMC8439011 34521469

[55] Jin C (2012). Human synovial lubricin expresses sialyl Lewis x determinant and has L-selectin ligand activity. J. Biol. Chem..

[56] Wang, N., Liang, H. & Zen, K. Molecular mechanisms that influence the macrophage M1-M2 polarization balance. *Front. Immunol.* **5**, 614 (2014). 10.3389/fimmu.2014.00614 PMC4246889 25506346

[57] Freitas MS (2016). Paracoccin induces M1 polarization of macrophages via interaction with TLR4. Front. Microbiol..

[58] Orr JS (2012). Toll-like receptor 4 deficiency promotes the alternative activation of adipose tissue macrophages. Diabetes.

[59] Suga H (2014). TLR4, rather than TLR2, regulates wound healing through TGF-β and CCL5 expression. J. Dermatol. Sci..

[60] Chen L, Guo S, Ranzer MJ, Dipietro LA (2013). Toll-like receptor 4 has an essential role in early skin wound healing. J. Invest. Dermatol..

[61] Schmidt TA, Schumacher BL, Klein TJ, Voegtline MS, Sah RL (2004). Synthesis of proteoglycan 4 by chondrocyte subpopulations in cartilage explants, monolayer cultures, and resurfaced cartilage cultures. Arthritis Rheum..

[62] Nakagawa, Y. et al. Cartilage derived from bone marrow mesenchymal stem cells expresses lubricin in vitro and in vivo. *PLoS ONE* **11**, e014877 (2016). 10.1371/journal.pone.0148777 PMC4750963 26867127

[63] Zhang C-H (2021). Creb5 establishes the competence for Prg4 expression in articular cartilage. Commun. Biol..

[64] Bechtold TE (2016). Excess BMP signaling in heterotopic cartilage Forming in Prg4 -null TMJ discs. J. Dent. Res..

[65] Mackay AM (1998). Chondrogenic differentiation of cultured human mesenchymal stem cells from marrow. Tissue Eng..

[66] Mwale F, Stachura D, Roughley P, Antoniou J (2006). Limitations of using aggrecan and type X collagen as markers of chondrogenesis in mesenchymal stem cell differentiation. J. Orthop. Res..

[67] Chavez, R. D., Sohn, P. & Serra, R. Prg4 prevents osteoarthritis induced by dominant-negative interference of TGF-ß signaling in mice. *PLoS ONE* **14**, e0210601 (2019). 10.1371/journal.pone.0210601 PMC6328116 30629676

[68] Jones ARC, Flannery CR (2007). Bioregulation of lubricin expression by growth factors and cytokines. Eur. Cell. Mater..

[69] Dong C, Zhu S, Wang T, Yoon W, Goldschmidt-Clermont PJ (2002). Upregulation of PAI-1 is mediated through TGF-β/Smad pathway in transplant arteriopathy. J. Hear. Lung Transpl..

[70] Omori, K. et al. Inhibition of plasminogen activator inhibitor- 1 attenuates transforming growth factor- β-dependent epithelial mesenchymal transition and differentiation of fibroblasts to myofibroblasts. *PLoS ONE* **11**, e0148969 (2016). 10.1371/journal.pone.0148969 PMC4747467 26859294

[71] Samarakoon R, Higgins SP, Higgins CE, Higgins PJ (2008). TGF-β1-induced plasminogen activator inhibitor-1 expression in vascular smooth muscle cells requires pp60c-src/EGFRY845 and Rho/ROCK signaling. J. Mol. Cell. Cardiol..

[72] He, Y. et al. LPS/TLR4 signaling enhances TGF-β response through downregulating BAMBI during prostatic hyperplasia. *Sci. Rep*. **6**, 27051 (2016). 10.1038/srep27051 PMC4886686 27243216

[73] Seki E (2007). TLR4 enhances TGF-β signaling and hepatic fibrosis. Nat. Med..

[74] Hurtig M (2019). Two compartment pharmacokinetic model describes the intra-articular delivery and retention of rhprg4 following ACL transection in the Yucatan mini pig. J. Orthop. Res..

[75] Abubacker S, McPeak A, Dorosz SG, Egberts P, Schmidt TA (2018). Effect of counterface on cartilage boundary lubricating ability by proteoglycan 4 and hyaluronan: cartilage-glass versus cartilage–cartilage. J. Orthop. Res..

[76] Miller D, Forrester K, Leonard C, Salo P, Bray RC (2005). ACL deficiency impairs the vasoconstrictive efficacy of neuropeptide Y and phenylephrine in articular tissues: a laser speckle perfusion imaging study. J. Appl. Physiol..

[77] Wilson AA (2013). Lentiviral delivery of RNAi for in vivo lineage-specific modulation of gene expression in mouse lung macrophages. Mol. Ther..

