# Supplementary material for: Proteoglycan 4 (PRG4) treatment enhances wound closure and tissue regeneration
Source: NPJ Regen Med. 2022 Jun 24;7:32. doi: 10.1038/s41536-022-00228-5 (PMC9232611; doi:10.1038/s41536-022-00228-5)
Supplement: Supplementary file 1 — Supplemental Figures [file 41536_2022_228_MOESM1_ESM.pdf]

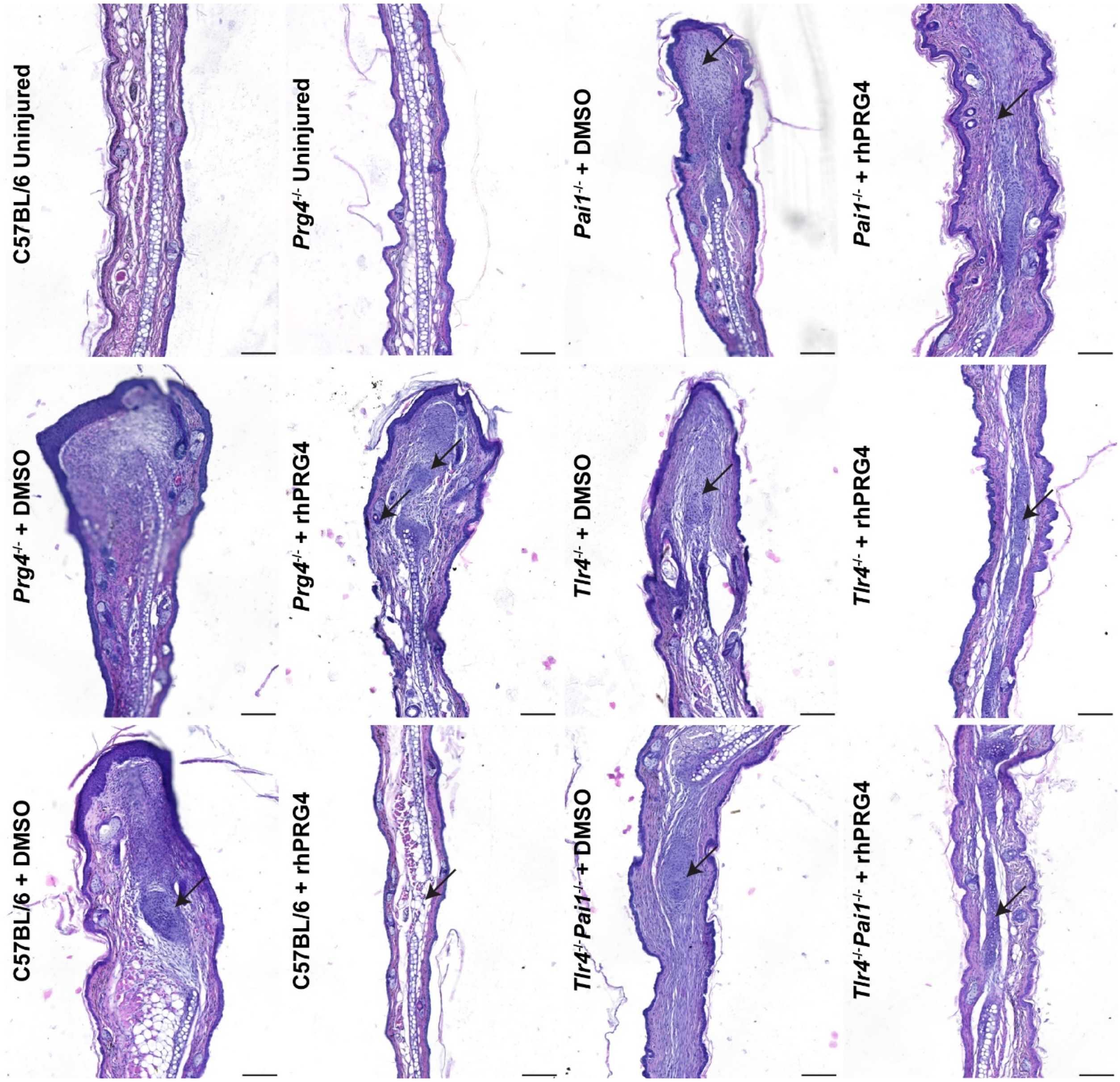

Supplementary Figure 1. Hematoxylin and Eosin staining of all mouse groups. To complement the Safranin O staining, H&E staining is provided here of all mice and treatment groups in the study at 4 weeks post-injury. Scale bar equals 50μm.

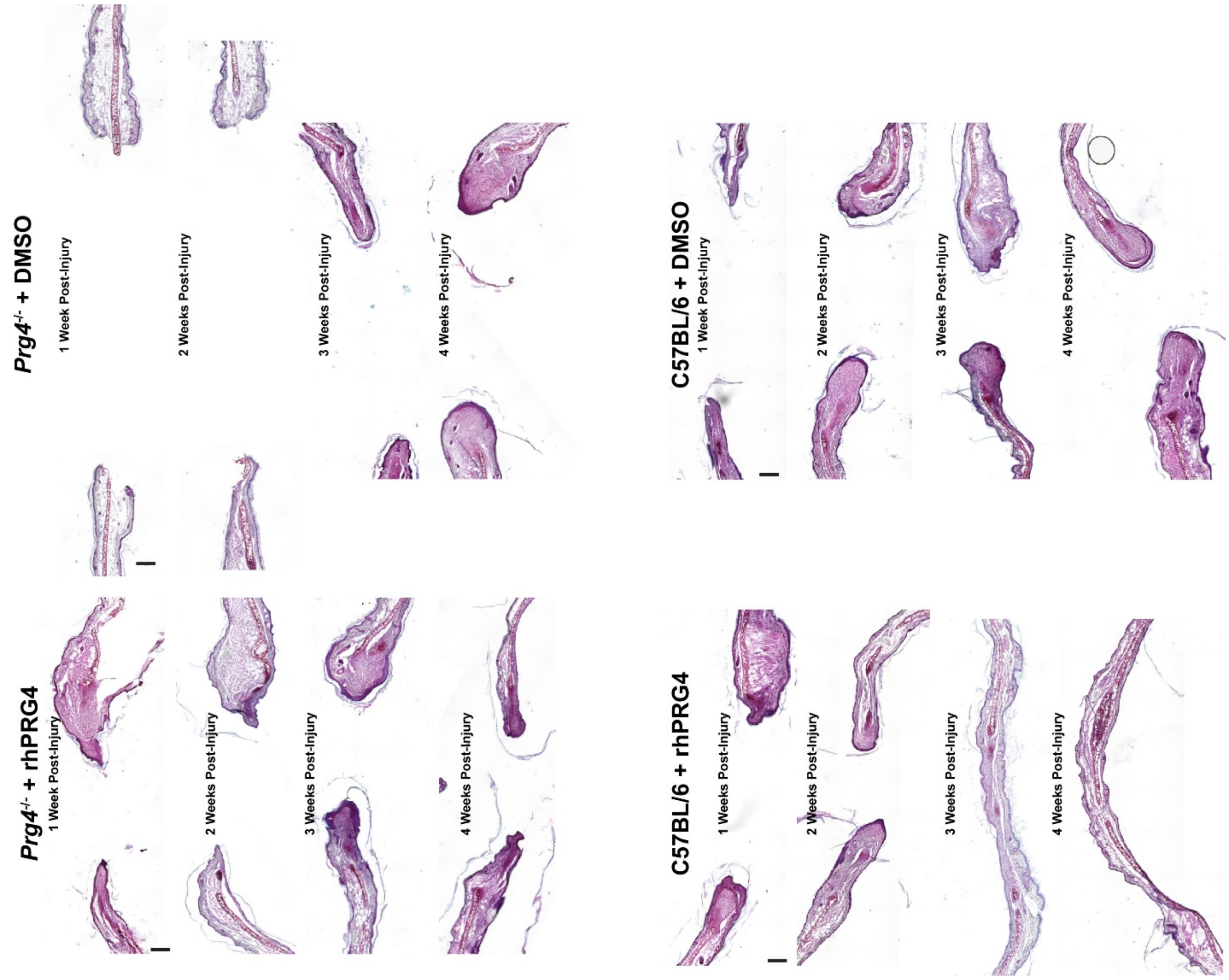

Supplementary Figure 2. Time course of healing in C57BL/6 and *Prg4*$^{-/-}$ mice with or without rhPRG4. Safranin O staining at each timepoint (1, 2, 3 and 4 weeks post-injury) of C57BL/6 and *Prg4*$^{-/-}$ mice with DMSO or rhPRG4 treatment. Please note that the *Prg4*$^{-/-}$ wounds treated with DMSO at 1 and 2 weeks post-injury did not fit within the objective field of view and were therefore compiled from two separate images. However, the distance between the tissue is accurate. Scale bar equals 50µm.

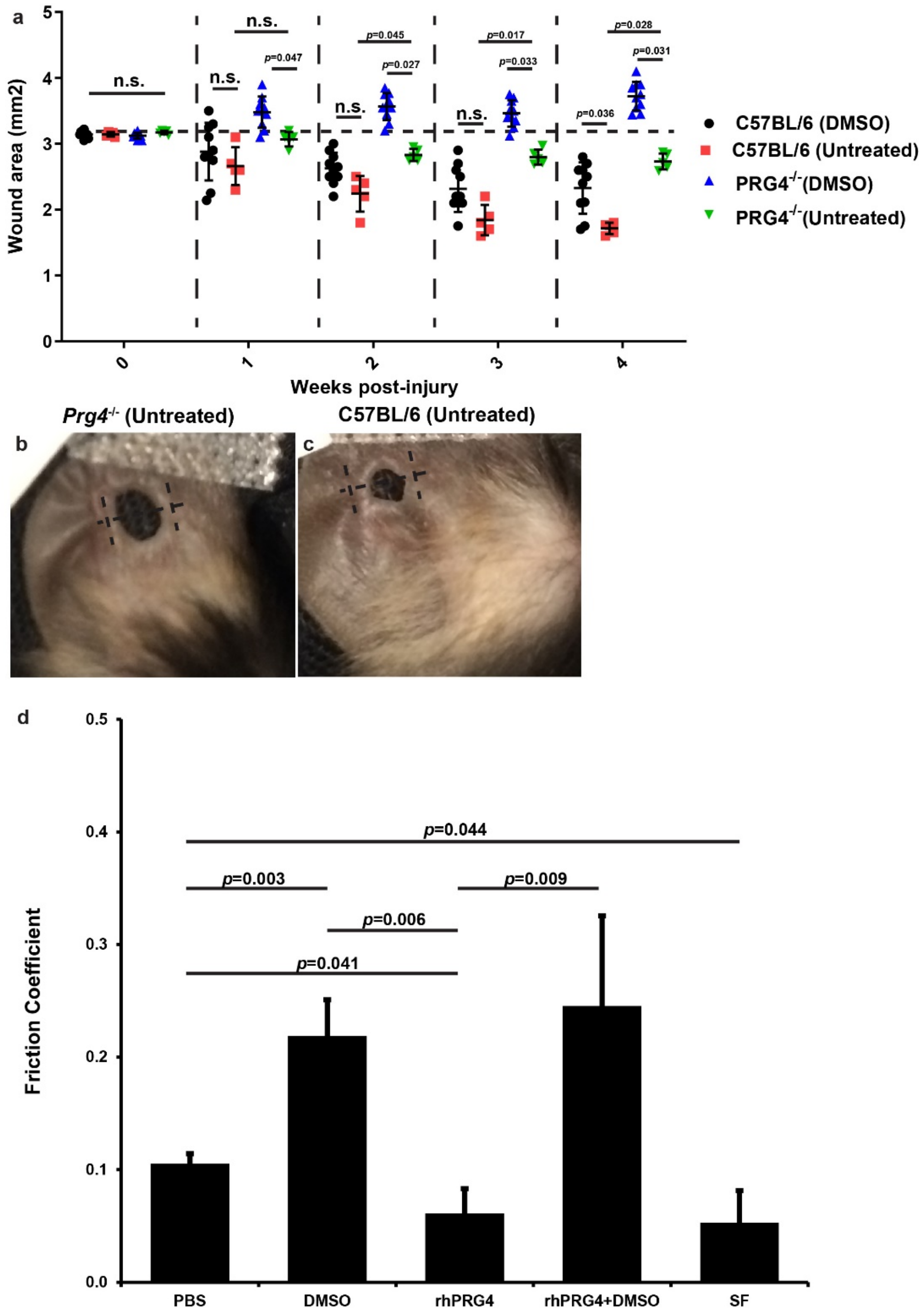

Supplementary Figure 3. Untreated injuries and friction testing of rhPRG4 post-treatment with DMSO. *Prg4*$^{-/-}$ and C57BL/6 mice were injured and left untreated (n=5 per group) and the rate of wound closure was compared to mice treated with DMSO (a,b). The kinetic friction coefficient of rhPRG4 (final concentration 100µg/ml) is lower than PBS and DMSO and is similar to that of human synovial fluid (SF). When rhPRG4 is mixed with DMSO (final concentration 100µg/ml), the resultant friction coefficient is similar to DMSO alone (b). All performed at 0.3 mm/s. Error bars equal mean +/- SD (a – 1 way ANNOVA, b – 2 way ANNOVA).

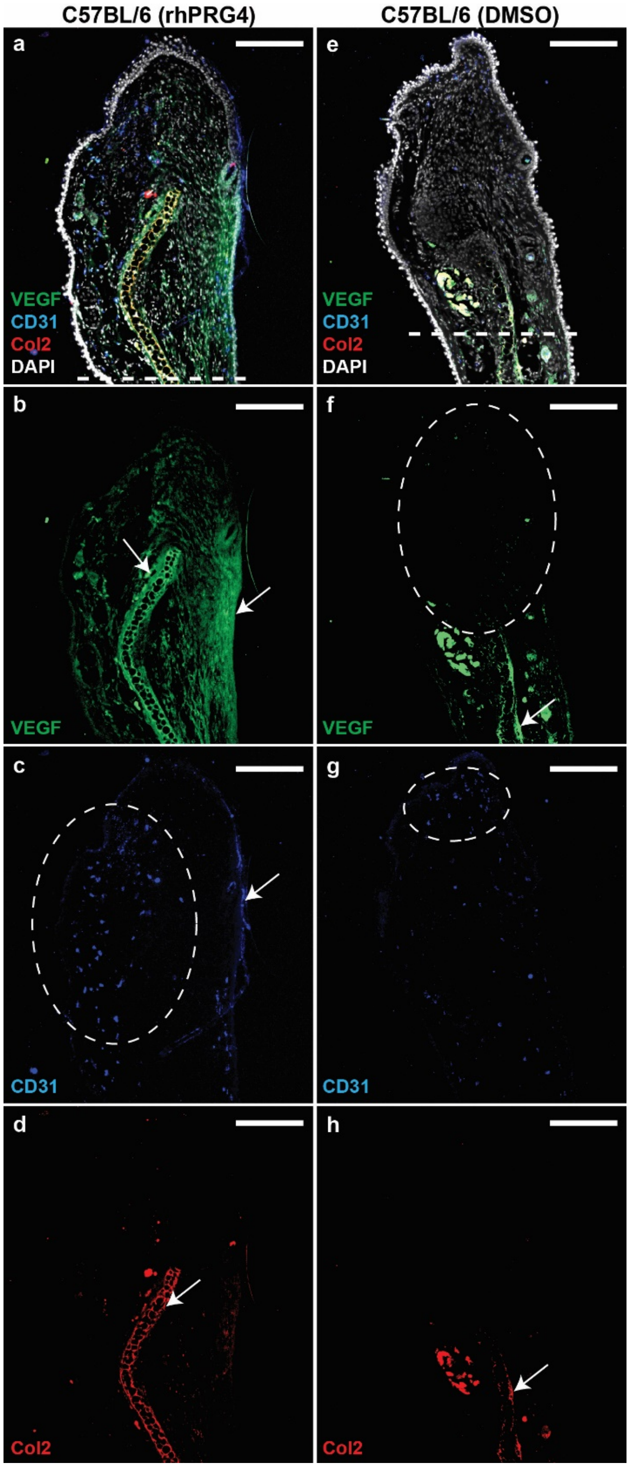

Supplementary Figure 4. VEGF and CD31 expression are increased in the wound area with rhPRG4 treatment. At 1 week post-injury, ears were stained for VEGF (green), CD31 (blue) and Collagen 2 (red). In the presence of rhPRG4 (a-d), increased VEGF, CD31 and Collagen 2 staining was observed vs. DMSO alone (e-h). While VEGF expression was observed throughout the injured ear with rhPRG4 treatment, it was colocalized with CD31 (arrows B,C) and Collagen 2 (b,d). In DMSO treated injuries, VEGF staining was nearly absent from the injury site (f) and only minimal CD31 staining was observed at the leading edge of the injury site (g). However, there was still colocalization observed between VEGF and Collagen 2 (arrow f,h) near the location of the original injury (dashed white line). Scale bar equals 50μm.

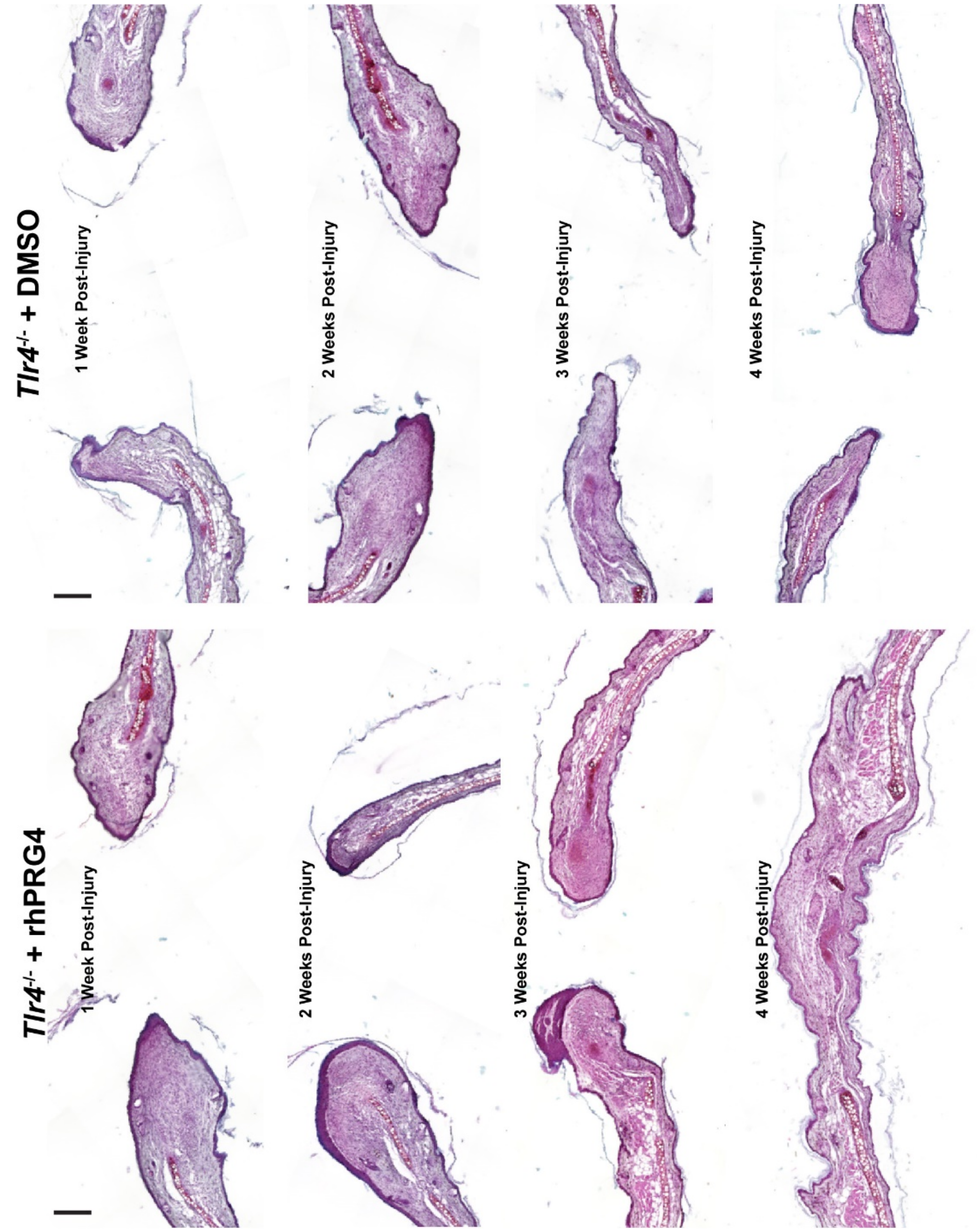

Supplementary Figure 5. Time course of healing in *Tlr4*$^{-/-}$ mice with or without rhPRG4. Safranin O staining at each timepoint (1, 2, 3 and 4 weeks post-injury) of *Tlr4*$^{-/-}$ mice with DMSO or rhPRG4 treatment. Scale bar equals 50μm.

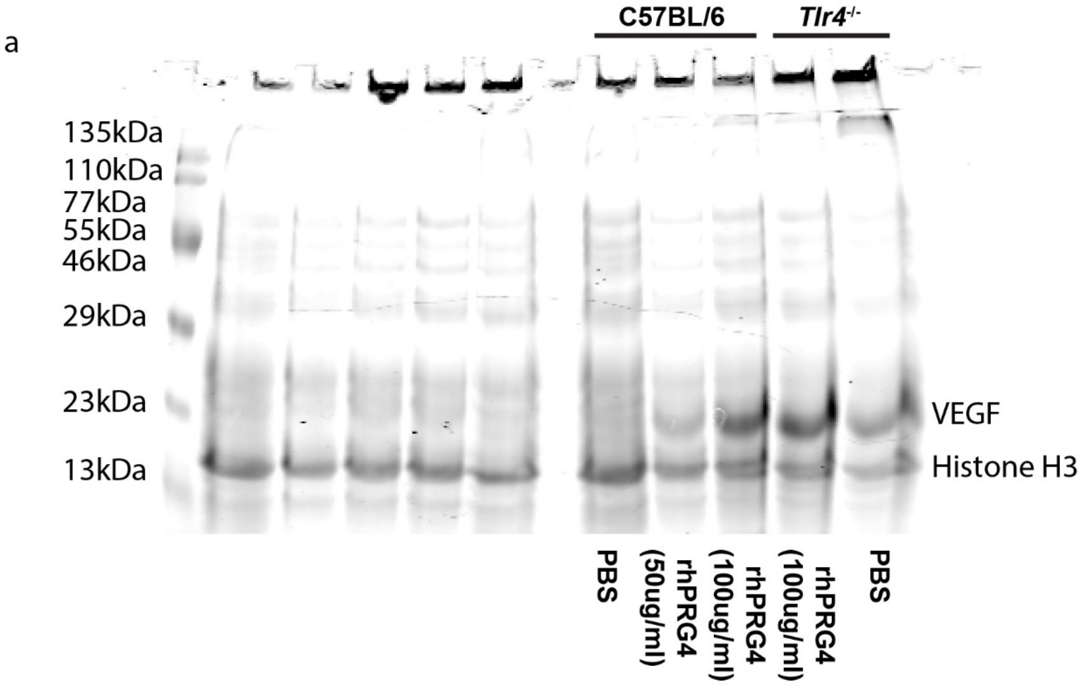

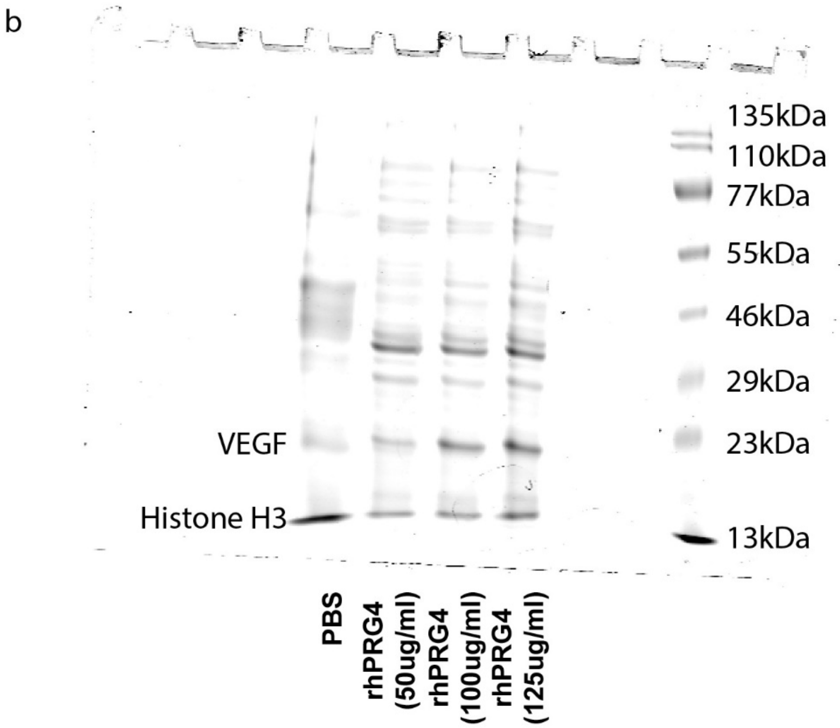

Supplementary Figure 6. Uncropped Western blots presented in the study.

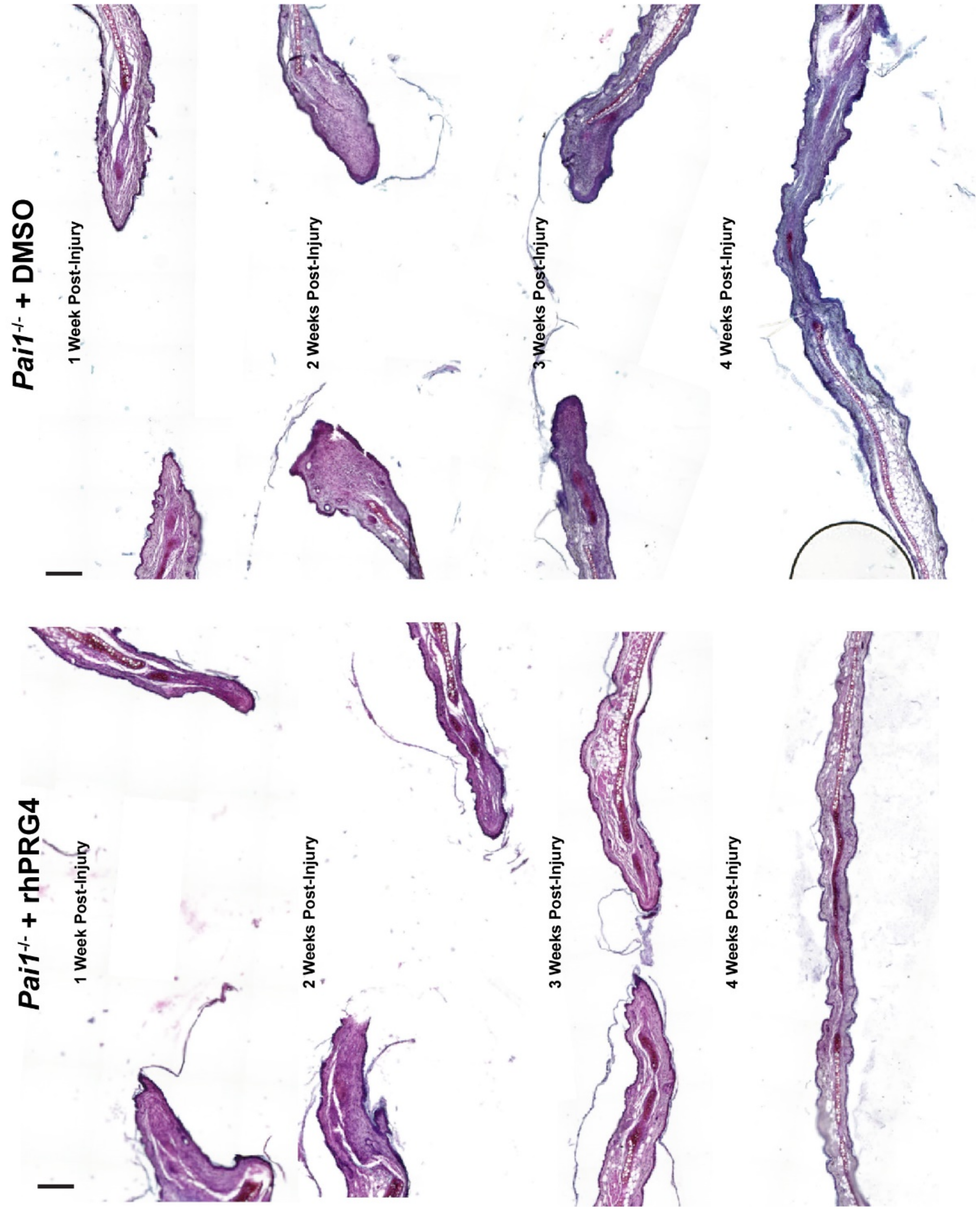

Supplementary Figure 7. Time course of healing in *Pai1*$^{-/-}$ mice with or without rhPRG4. Safranin O staining at each timepoint (1, 2, 3 and 4 weeks post-injury) of *Pai1*$^{-/-}$ mice with DMSO or rhPRG4 treatment. Scale bar equals 50µm.

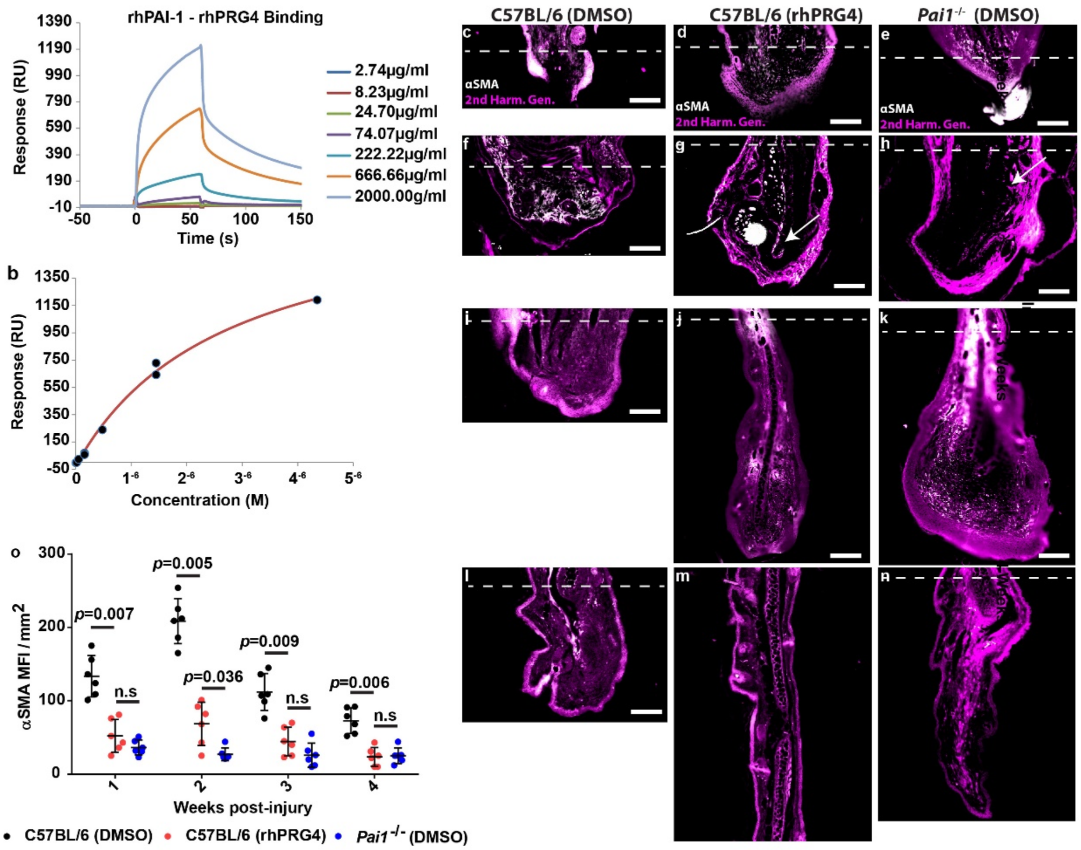

Supplementary Figure 8. PAI1 binds to rhPRG4 and regulates fibrosis. SPR analysis demonstrates that PAI1 binds directly to rhPRG4 (a,b), with a binding constant of 2.712×10^{-6}M. The level of the fibrotic marker αSMA was quantified in the ear tissue surrounding the injury site and representative images are presented (c-o). Second harmonic generation is shown in pink, while αSMA is shown in white. The dashed white line represents initial injury site. Scale bar equals 50µm. n.s. = not significant. Error bars equal mean +/- SD (o – 1 way ANNOVA).

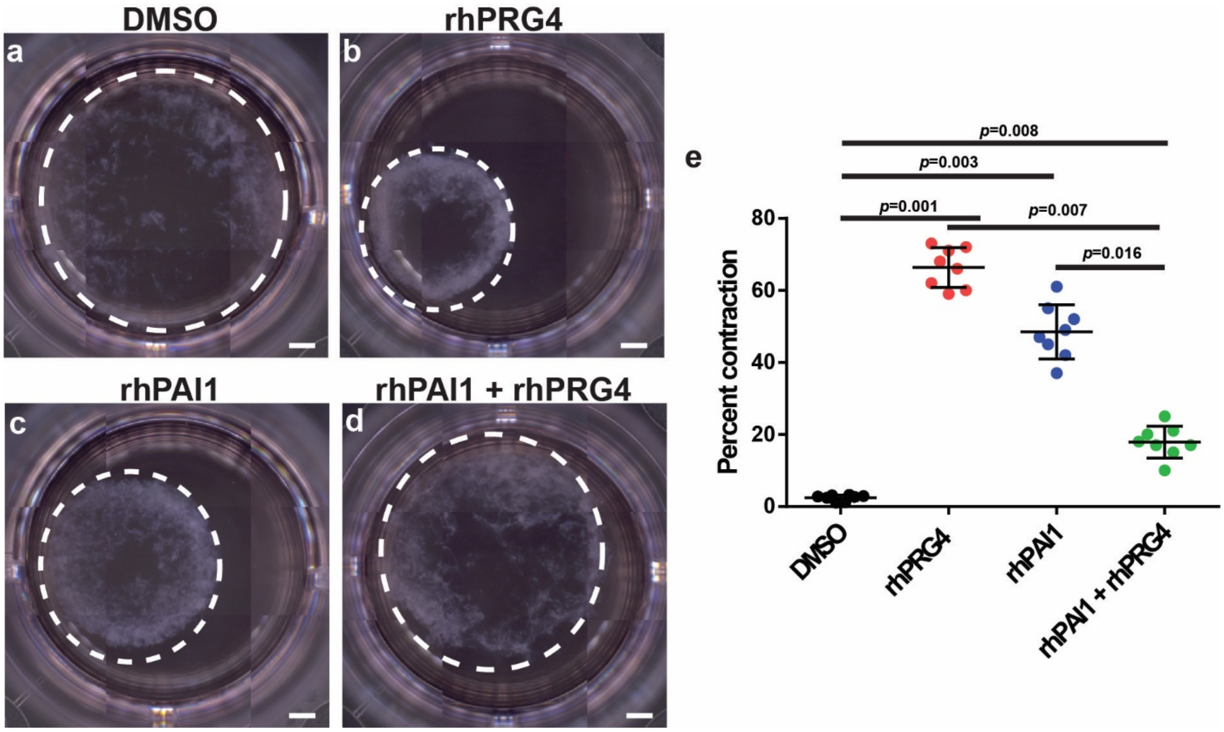

Supplementary Figure 9. rhPRG4 or PAI1 induce contraction, while the combination inhibits contraction. Collagen gel contraction assays demonstrated that both rhPRG4 or PAI1 induce collagen contraction vs. DMSO alone (a-c,e). However, when rhPRG4 and PAI1 were mixed together, collagen contraction was inhibited (d,e). Scale bar equals 100µm. Error bars equal mean +/- SD (e – 1 way ANNOVA).

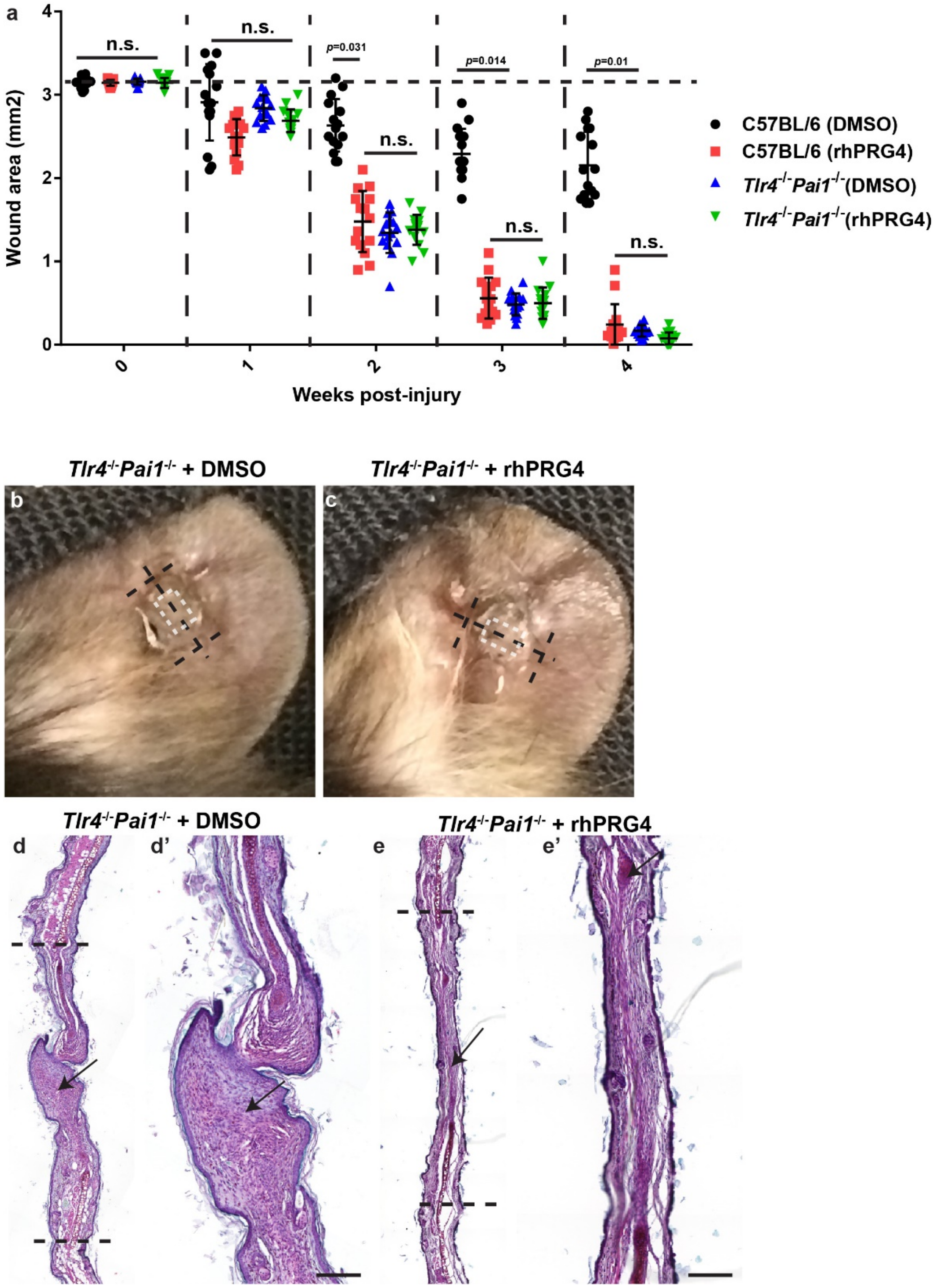

Supplementary Figure 10. *Tlr4*$^{-/-}$*Pai1*$^{-/-}$ mice demonstrate increased wound healing post-injury. *Tlr4*$^{-/-}$*Pai1*$^{-/-}$ mice demonstrate increased wound closure compared to C57BL/6 mice and this was not enhanced in the presence of rhPRG4 (a). Representative images of ears 4 weeks post-injury (b,c). The black dashed line represents the plane of histological sectioning which the original injury diameter, while the white dashed box indicates the location of the histological image presented (b,c). Representative histological images stained with Safranin O, 4 weeks post-injury (d,e) with the dashed black line representing the original injury site. Minimal new mature cartilage can be observed in *Tlr4*$^{-/-}$*Pai1*$^{-/-}$ mice treated with DMSO (arrows, d), or in the presence of rhPRG4 (arrow, e). Scale bar equals 50µm. n.s. = not significant. Error bars equal mean +/- SD (A – 2 way ANNOVA).

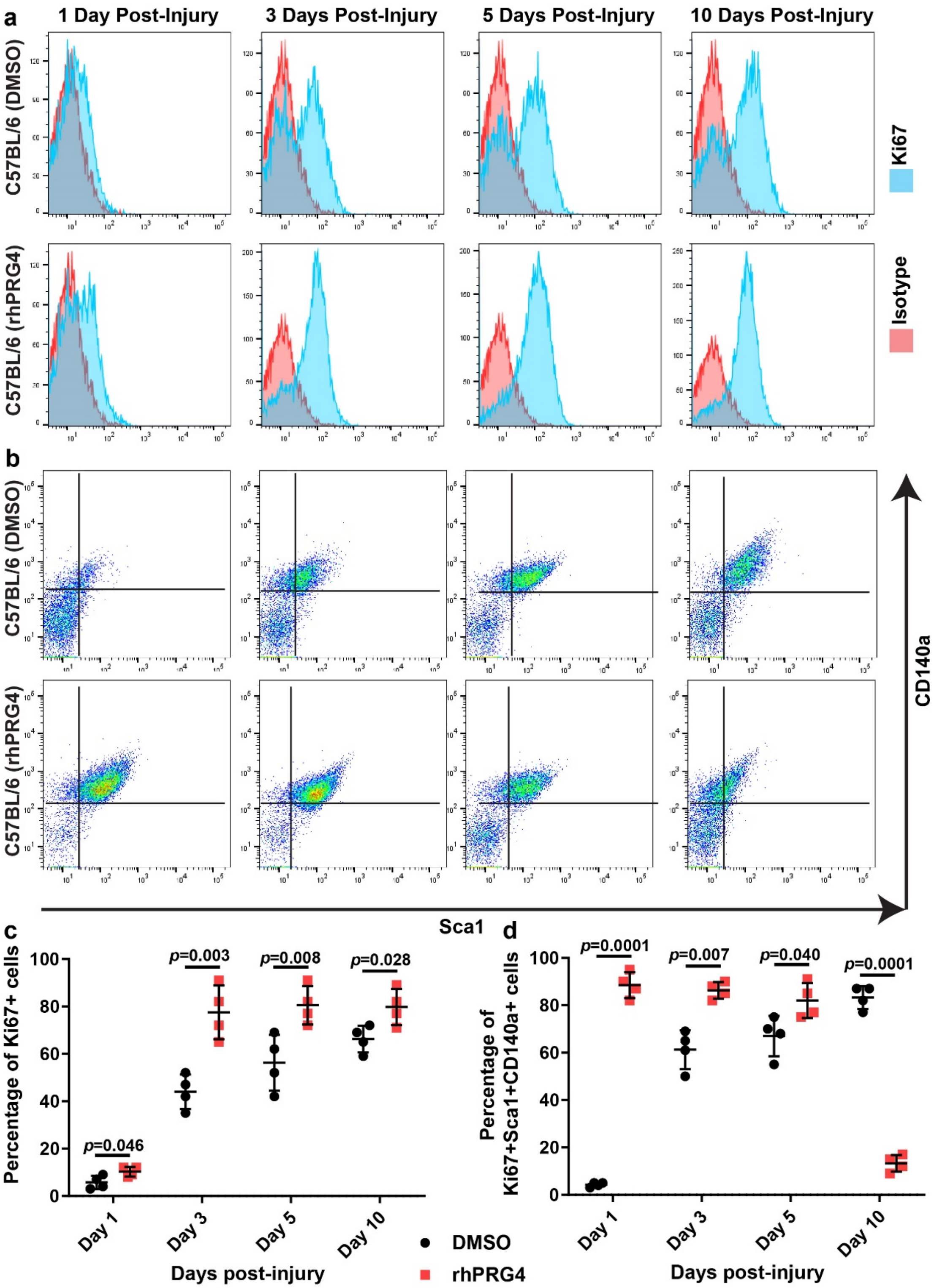

Supplementary Figure 11. Effects of rhPRG4 on chondrogenesis *in vivo*. Post-injury, *Hic1*$^{+}$ progenitors give rise to Collagen II^{+} cells within the injury site (a) and this effect in more pronounced in wounds treated with rhPRG4 vs. DMSO (b). In vivo experiments were undertaken on n=3 animals per group. Bars represent mean with SD. *** = p<0.001 (t-test).

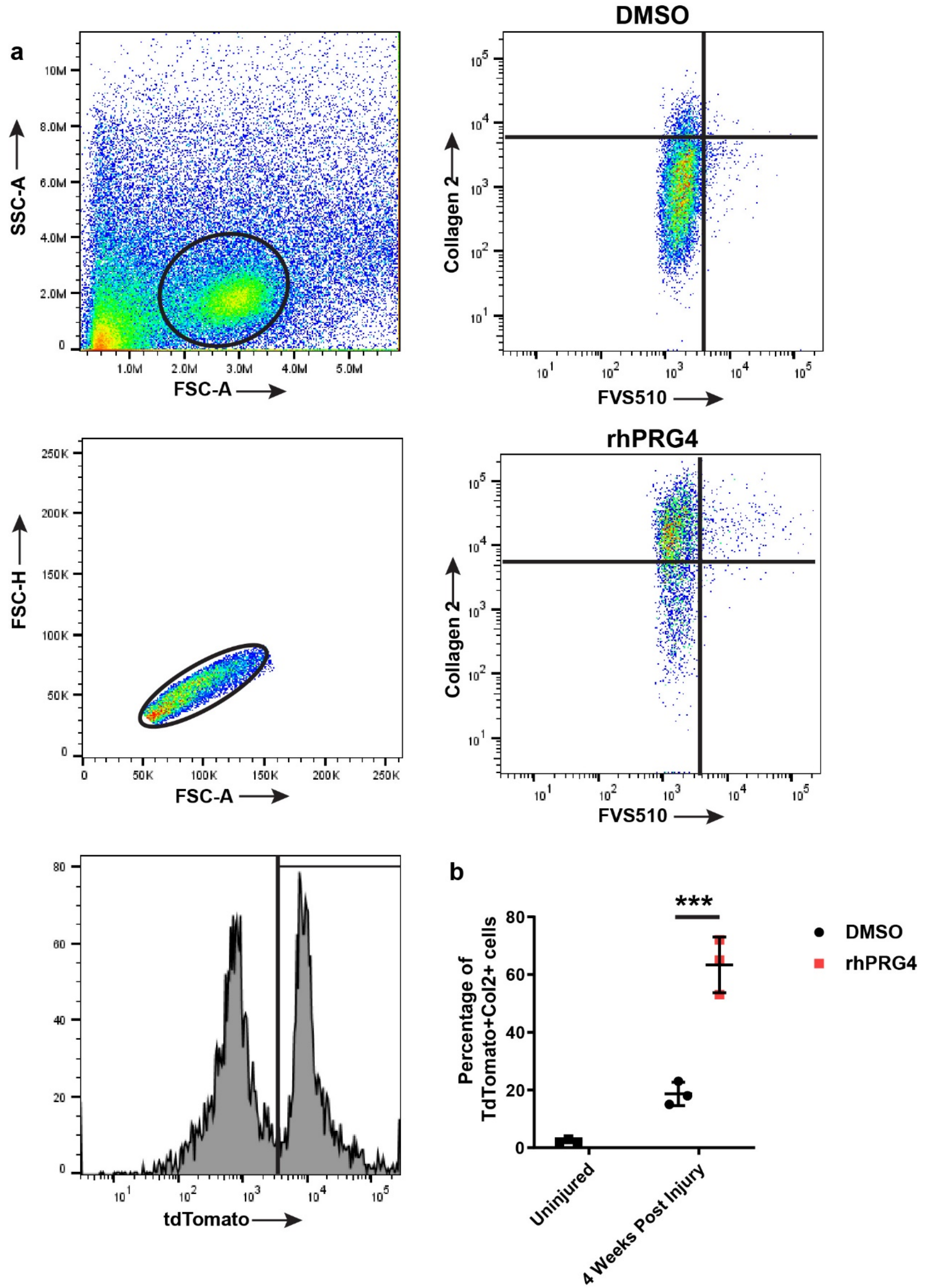

Supplementary Figure 12. Gating strategy for MPCs and macrophages. Representative examples of flow cytometry gating strategies for MPCs and macrophage populations.

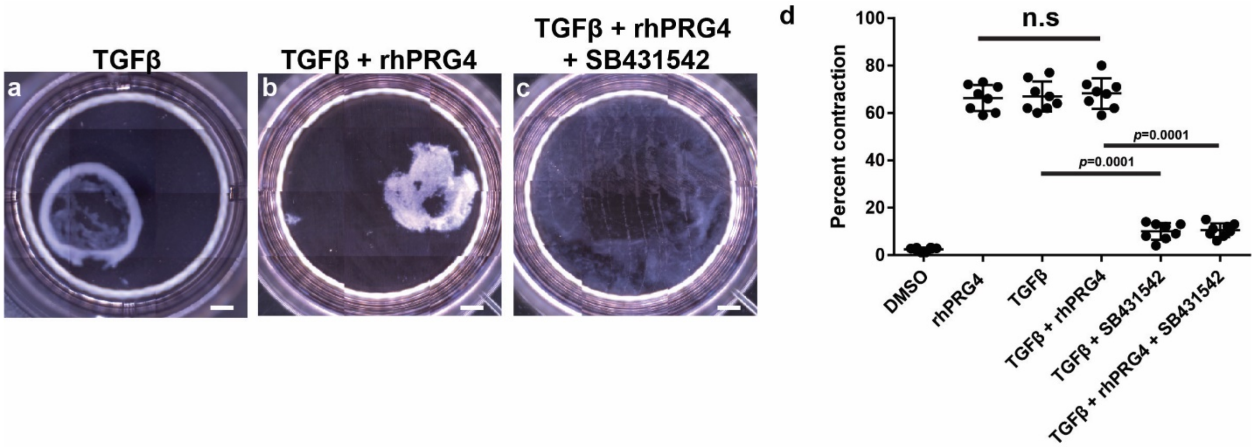

Supplementary Figure 13. Inhibition of the TGFβ pathway inhibits rhPRG4 induced contraction. TGFβ was found to induce collagen gel contraction (a,d), and this effect was not synergistic with rhPRG4 (b,d). However, inhibition of the TGFβ pathway with SB431541 abrogated both TGFβ and rhPRG4 induced collagen gel contraction (c,d). Scale bar equals 100μm. n.s. = not significant. Error bars equal mean +/- SD (D – 1 way ANNOVA).

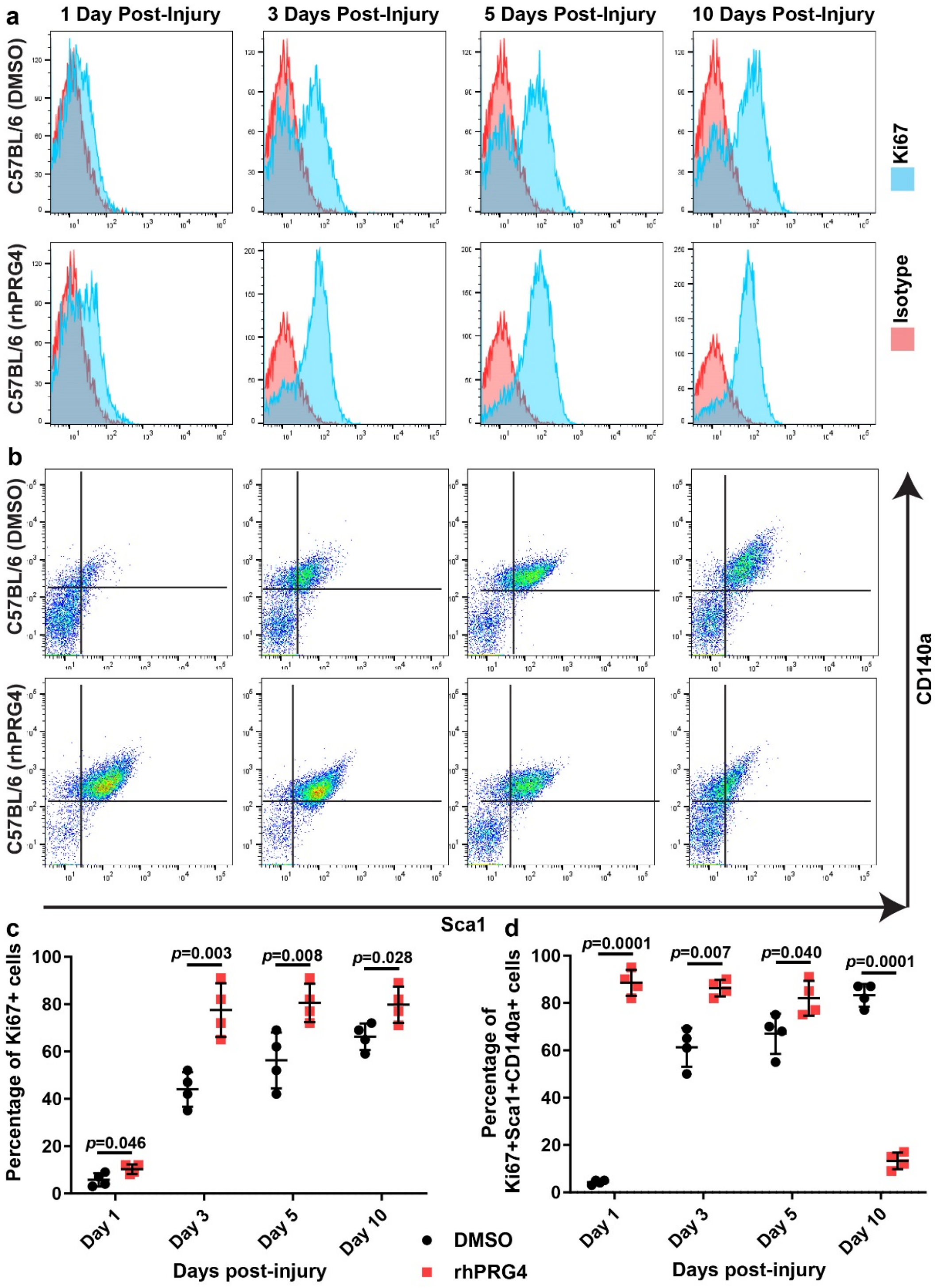

Supplementary Figure 14. Proliferation in injury associated cells and MPCs with rhPRG4 treatment. A 3mm secondary biopsy was generated around the primary 2mm wound injury at various timepoints post-injury and the cells were examined for Ki67 expression in the total cell population (a,c) as well as the MPC ($Sca1^{+}CD140^{+}$) sub-population (b,d). All experiments were undertaken on at least 3 biological and 3 technical replicates unless otherwise stated. n.s. = not significant. Error bars equal mean +/- SD (c,d – 1 way ANNOVA).

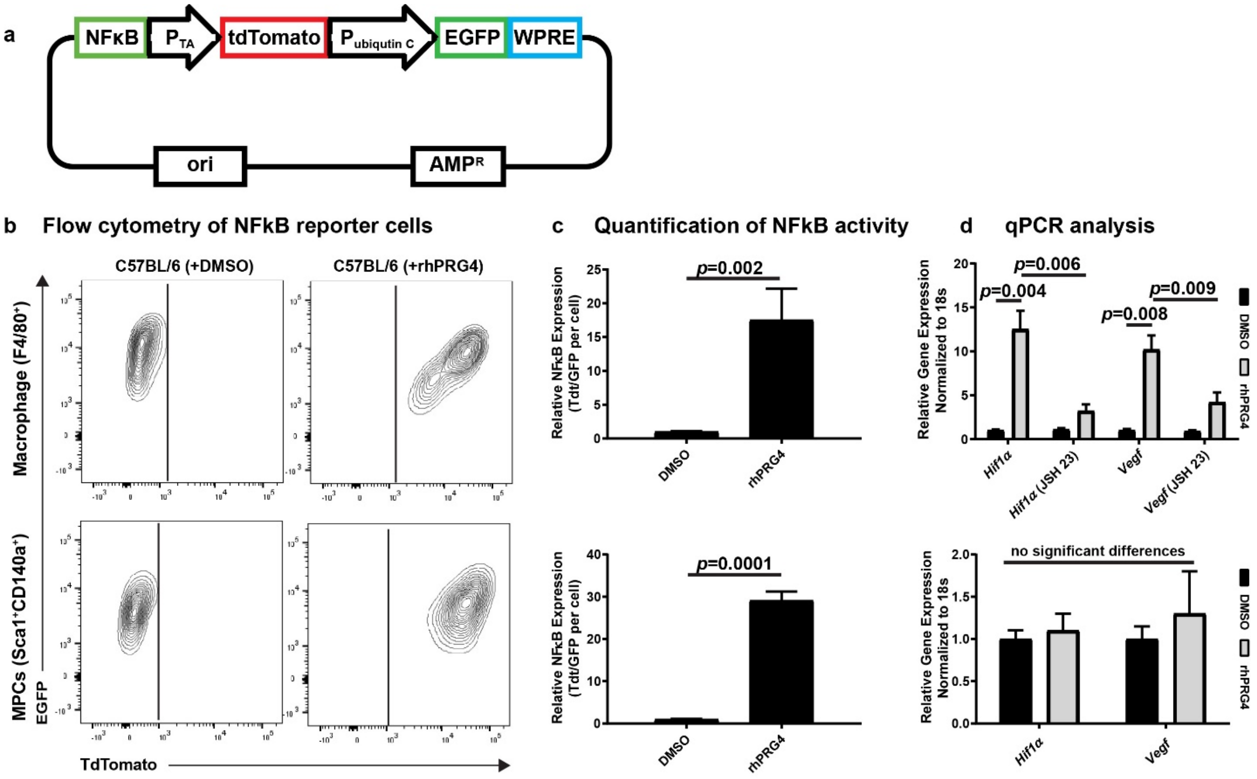

Supplementary Figure 15. rhPR4G4 treatment of macrophages induced *Vegf* through an NFκB dependent pathway. An NFκB reporter plasmid was generated in where NFκB activation is tied to tdTomato expression and EGFP expression is expressed in all cells that have taken up the plasmid (a). Macrophages (F4/80^{+}) and MPCs (Sca1^{+}CD140a^{+}) were gated on positive EGFP expression and exposed to rhPRG4 (b). Both macrophages and MPCs activated the NFκB pathway in response to rhPRG4 (c). However, only macrophages increased the expression of *Hif1α* and *Vegf* in response to rhPRG4 (d). In macrophages, the activation of *Hif1α* and *Vegf* was inhibited in the presence of the NFκB inhibitor (JSH 23) (d). All experiments were undertaken on at least 3 biological and 3 technical replicates unless otherwise stated. ***p<0.001; **p<0.01; n.s. = not significant. Error bars equal mean +/- SD (c,d – t-test).
