# Supplementary material for: Proteoglycan 4 (PRG4) treatment enhances wound closure and tissue regeneration
Source: NPJ Regen Med. 2022 Jun 24;7:32. doi: 10.1038/s41536-022-00228-5 (PMC9232611; doi:10.1038/s41536-022-00228-5)
Supplement: Supplementary file 2 — REPORTING SUMMARY [file 41536_2022_228_MOESM2_ESM.pdf]

nature portfolio

Corresponding author(s): Roman Krawetz

Last updated by author(s): Jan 7, 2022

# Reporting Summary

Nature Portfolio wishes to improve the reproducibility of the work that we publish. This form provides structure for consistency and transparency in reporting. For further information on Nature Portfolio policies, see our Editorial Policies and the Editorial Policy Checklist.

## Statistics

For all statistical analyses, confirm that the following items are present in the figure legend, table legend, main text, or Methods section.

| n/a | Confirmed | |
|---|---|---|
| ☐ | ☒ | The exact sample size ($n$) for each experimental group/condition, given as a discrete number and unit of measurement |
| ☐ | ☒ | A statement on whether measurements were taken from distinct samples or whether the same sample was measured repeatedly |
| ☐ | ☒ | The statistical test(s) used AND whether they are one- or two-sided<br>*Only common tests should be described solely by name; describe more complex techniques in the Methods section.* |
| ☒ | ☐ | A description of all covariates tested |
| ☐ | ☒ | A description of any assumptions or corrections, such as tests of normality and adjustment for multiple comparisons |
| ☐ | ☒ | A full description of the statistical parameters including central tendency (e.g. means) or other basic estimates (e.g. regression coefficient) AND variation (e.g. standard deviation) or associated estimates of uncertainty (e.g. confidence intervals) |
| ☐ | ☒ | For null hypothesis testing, the test statistic (e.g. $F$, $t$, $r$) with confidence intervals, effect sizes, degrees of freedom and $P$ value noted<br>*Give P values as exact values whenever suitable.* |
| ☒ | ☐ | For Bayesian analysis, information on the choice of priors and Markov chain Monte Carlo settings |
| ☒ | ☐ | For hierarchical and complex designs, identification of the appropriate level for tests and full reporting of outcomes |
| ☒ | ☐ | Estimates of effect sizes (e.g. Cohen's $d$, Pearson's $r$), indicating how they were calculated |

*Our web collection on statistics for biologists contains articles on many of the points above.*

## Software and code

Policy information about availability of computer code

Data collection: no software was used for data collection

Data analysis: statistical data was analyzed using GraphPad Prism 6 software. Flow cyomtery data was analyzed using flowjo .

For manuscripts utilizing custom algorithms or software that are central to the research but not yet described in published literature, software must be made available to editors and reviewers. We strongly encourage code deposition in a community repository (e.g. GitHub). See the Nature Portfolio guidelines for submitting code & software for further information.

## Data

Policy information about availability of data

All manuscripts must include a data availability statement. This statement should provide the following information, where applicable:

- Accession codes, unique identifiers, or web links for publicly available datasets
- A description of any restrictions on data availability
- For clinical datasets or third party data, please ensure that the statement adheres to our policy

All data are available in the main text or the supplementary materials.

# Field-specific reporting

Please select the one below that is the best fit for your research. If you are not sure, read the appropriate sections before making your selection.

☒ Life sciences ☐ Behavioural & social sciences ☐ Ecological, evolutionary & environmental sciences

For a reference copy of the document with all sections, see nature.com/documents/nr-reporting-summary-flat.pdf

# Life sciences study design

All studies must disclose on these points even when the disclosure is negative.

| | |
|---|---|
| Sample size | Samples were based on preliminary data and previously published studies from your groups and others. |
| Data exclusions | no data was excluded |
| Replication | regardless of sample size, all data presented in the study was at least independently repeated once with similar outcomes obtained. |
| Randomization | all animals were randomly assigned to groups, except for sex which was kept as equal as possible between groups. |
| Blinding | blinding was employed whenever possible in the study. |

# Reporting for specific materials, systems and methods

We require information from authors about some types of materials, experimental systems and methods used in many studies. Here, indicate whether each material, system or method listed is relevant to your study. If you are not sure if a list item applies to your research, read the appropriate section before selecting a response.

## Materials & experimental systems

| n/a | Involved in the study | |
|---|---|---|
| ☐ | ☒ | Antibodies |
| ☐ | ☒ | Eukaryotic cell lines |
| ☒ | ☐ | Palaeontology and archaeology |
| ☐ | ☒ | Animals and other organisms |
| ☒ | ☐ | Human research participants |
| ☒ | ☐ | Clinical data |
| ☒ | ☐ | Dual use research of concern |

## Methods

| n/a | Involved in the study | |
|---|---|---|
| ☒ | ☐ | ChIP-seq |
| ☐ | ☒ | Flow cytometry |
| ☒ | ☐ | MRI-based neuroimaging |

## Antibodies

| | |
|---|---|
| Antibodies used | Primary antibodies (VEGF [clone # VG1 – Invitrogen], CD31 [clone #390 – Invitrogen, Col2 [clone # II-II6B3 – DSHB, Iowa], αSMA [clone # 1A4 – ABCAM] and PRG4 [clone # 9G3 – Millipore]), Histone H3 (Rabbit polyclonal – Invitrogen), Sca-1 (clone D7), CD140 (clone APA5), both ThermoFisher. F4/80 (clone BM8), CD11b (clone M1/70), both ThermoFisher. |
| Validation | Our groups have used and published on all these antibodies previously. And they were originally selected based on previous publications in the field. |

## Eukaryotic cell lines

Policy information about cell lines

| | |
|---|---|
| Cell line source(s) | All cells used in the current study are primary cells that were derived by our group. |
| Authentication | Cells used were isolated/dervied by gold standard methods commonly used in the field and validated by the expression of cell surface proteins by flow cytometry. |
| Mycoplasma contamination | The cells used in this study were not tested for mycoplasma |
| Commonly misidentified lines (See ICLAC register) | n/a |

## Animals and other organisms

Policy information about studies involving animals; ARRIVE guidelines recommended for reporting animal research

| | |
|---|---|
| Laboratory animals | For in vivo experiments, 10-week-old mice were used, with equal numbers of males and females in each group. Animal size sample was determined by power analysis based on preliminary data. Mice were randomized across treatment groups. |
| Wild animals | the study did not use wild animals |
| Field-collected samples | no field collected animals/samples were used |
| Ethics oversight | This study was carried out in accordance with the recommendations in the Canadian Council on Animal Care Guidelines. Animal protocols and surgical procedures were approved by the University of Calgary Animal Care Committee (protocol AC16-0043). All surgery was performed under isoflurane anaesthesia. Number of repeats is specified in each figure legend. |

Note that full information on the approval of the study protocol must also be provided in the manuscript.

## Flow Cytometry

### Plots

Confirm that:

☒ The axis labels state the marker and fluorochrome used (e.g. CD4-FITC).

☒ The axis scales are clearly visible. Include numbers along axes only for bottom left plot of group (a 'group' is an analysis of identical markers).

☒ All plots are contour plots with outliers or pseudocolor plots.

☒ A numerical value for number of cells or percentage (with statistics) is provided.

### Methodology

| | |
|---|---|
| Sample preparation | In experiments where RNA was collected and/or single cells were required for cell sorting/flow cytometry; the ear tissue surrounding the injury area was removed using a 3mm biopsy punch and the resultant tissue was dissociated using the Multi Tissue Dissociation Kit 1 (Miltenyi Biotec), following the Dissociation of mouse ear following the Multi Tissue Dissociation Kit 1 protocol. |
| Instrument | BD FACS Aria Fusion (BD Biosciences) |
| Software | Data was collected using BD FACSDiva™. Analysis was done using FlowJo. |
| Cell population abundance | Purity was determined by running a purity check of the sorted populations after the sort was completed. |
| Gating strategy | All samples were gated based on forward and side scatter to identify cellular events. Forward scatter height vs. area to identify single cells. Alive cells were selected by negativity for viability dye. All following gaiting steps are presented in principal and supplementary figures. |

☒ Tick this box to confirm that a figure exemplifying the gating strategy is provided in the Supplementary Information.
